# Genome analysis of a major urban malaria vector mosquito, *Anopheles stephensi*

**DOI:** 10.1186/s13059-014-0459-2

**Published:** 2014-09-23

**Authors:** Xiaofang Jiang, Ashley Peery, A Brantley Hall, Atashi Sharma, Xiao-Guang Chen, Robert M Waterhouse, Aleksey Komissarov, Michelle M Riehle, Yogesh Shouche, Maria V Sharakhova, Dan Lawson, Nazzy Pakpour, Peter Arensburger, Victoria L M Davidson, Karin Eiglmeier, Scott Emrich, Phillip George, Ryan C Kennedy, Shrinivasrao P Mane, Gareth Maslen, Chioma Oringanje, Yumin Qi, Robert Settlage, Marta Tojo, Jose M C Tubio, Maria F Unger, Bo Wang, Kenneth D Vernick, Jose M C Ribeiro, Anthony A James, Kristin Michel, Michael A Riehle, Shirley Luckhart, Igor V Sharakhov, Zhijian Tu

**Affiliations:** Program of Genetics, Bioinformatics, and Computational Biology, Virginia Tech, Blacksburg, VA USA; Department of Biochemistry, Virginia Tech, Blacksburg, VA USA; Department of Entomology, Virginia Tech, Blacksburg, VA USA; Department of Pathogen Biology, Southern Medical University, Guangzhou, Guangdong China; Department of Genetic Medicine and Development, University of Geneva Medical School, rue Michel-Servet 1, 1211 Geneva, Switzerland; Swiss Institute of Bioinformatics, rue Michel-Servet 1, 1211 Geneva, Switzerland; Computer Science and Artificial Intelligence Laboratory, Massachusetts Institute of Technology, 32 Vassar Street, Cambridge, MA USA; The Broad Institute of MIT and Harvard, 7 Cambridge Center, Cambridge, MA USA; Theodosius Dobzhansky Center for Genome Bioinformatics, St. Petersburg State University, St. Petersburg, Russia; Institute of Cytology Russian Academy of Sciences, St. Petersburg, Russia; Department of Microbiology, University of Minnesota, Minneapolis, MN USA; National Center for Cell Science, Pune University Campus, Ganeshkhind, Pune India; European Molecular Biology Laboratory, European Bioinformatics Institute, Wellcome Trust Genome Campus, Hinxton, Cambridge CB10 1SD UK; Department of Medical Microbiology and Immunology, University of California, Davis, CA USA; Biological Sciences Department, California State Polytechnic University, Pomona, CA USA; Division of Biology, Kansas State University, Manhattan, KS USA; Department of Parasitology and Mycology, Unit of Insect Vector Genetics and Genomics, Institut Pasteur, Paris, France; CNRS Unit of Hosts, Vectors and Pathogens (URA3012), Paris, France; Department of Computer Science and Engineering, University of Notre Dame, Notre Dame, IN USA; Department of Bioengineering and Therapeutic Sciences, University of California, San Francisco, CA USA; Virginia Bioinformatics Institute, Virginia Tech, Blacksburg, VA USA; Department of Entomology, University of Arizona, Tucson, AZ USA; Department of Physiology, School of Medicine – CIMUS, Instituto de Investigaciones Sanitarias, University of Santiago de Compostela, Santiago de Compostela, Spain; Wellcome Trust Sanger Institute, Hinxton, Cambridgeshire UK; Department of Biological Sciences, University of Notre Dame, Notre Dame, IN USA; Section of Vector Biology, Laboratory of Malaria and Vector Research, National Institute of Allergy and Infectious Diseases, Rockville, MD USA; Departments of Microbiology & Molecular Genetics and Molecular Biology & Biochemistry, University of California, Irvine, CA USA

## Abstract

**Background:**

*Anopheles stephensi* is the key vector of malaria throughout the Indian subcontinent and Middle East and an emerging model for molecular and genetic studies of mosquito-parasite interactions. The type form of the species is responsible for the majority of urban malaria transmission across its range.

**Results:**

Here, we report the genome sequence and annotation of the Indian strain of the type form of *An. stephensi*. The 221 Mb genome assembly represents more than 92% of the entire genome and was produced using a combination of 454, Illumina, and PacBio sequencing. Physical mapping assigned 62% of the genome onto chromosomes, enabling chromosome-based analysis. Comparisons between *An. stephensi* and *An. gambiae* reveal that the rate of gene order reshuffling on the X chromosome was three times higher than that on the autosomes. *An. stephensi* has more heterochromatin in pericentric regions but less repetitive DNA in chromosome arms than *An. gambiae*. We also identify a number of Y-chromosome contigs and BACs. Interspersed repeats constitute 7.1% of the assembled genome while LTR retrotransposons alone comprise more than 49% of the Y contigs. RNA-seq analyses provide new insights into mosquito innate immunity, development, and sexual dimorphism.

**Conclusions:**

The genome analysis described in this manuscript provides a resource and platform for fundamental and translational research into a major urban malaria vector. Chromosome-based investigations provide unique perspectives on *Anopheles* chromosome evolution. RNA-seq analysis and studies of immunity genes offer new insights into mosquito biology and mosquito-parasite interactions.

**Electronic supplementary material:**

The online version of this article (doi:10.1186/s13059-014-0459-2) contains supplementary material, which is available to authorized users.

## Background

Mosquitoes in the genus *Anopheles* are the primary vectors of human malaria parasites and the resulting disease is one of the most deadly and costly in history [1,2]. Publication and availability of the *Anopheles gambiae* genome sequence accelerated research that has not only enhanced our basic understanding of vector genetics, behavior, and physiology and roles in transmission, but also contributed to new strategies for combating malaria [3]. Recent application of next-generation sequencing technologies to mosquito genomics offers exciting opportunities to expand our understanding of mosquito biology in many important vector species and harness the power of comparative genomics. Such information will further facilitate the development of new strategies to combat malaria and other mosquito-borne diseases. *An. stephensi* is among approximately 60 species considered important in malaria transmission and is the key vector of urban malaria on the Indian subcontinent and the Middle East [4,5]. The fact that a recent resurgence of human malaria in Africa could have been caused by the sudden appearance of *An. stephensi* indicates that *An. stephensi* may pose an even greater risk to human health in the future [6]. Of the three forms, type, *mysorensis*, and intermediate, the former is responsible for the majority, if not all, of urban malaria transmission across its range and accounts for approximately 12% of all transmission in India [7]. Thus efforts to control it can be expected to contribute significantly to the malaria eradication agenda [8,9]. *An. stephensi* is amenable to genetic manipulations such as transposon-based germline transformation [10], genome-wide mutagenesis [11], site-specific integration [12], genome-editing [13], and RNAi-based functional genomics analysis [14]. Our understanding of the interactions between *An. stephensi* and the malaria parasites is rapidly improving [15-20]. Thus *An. stephensi* is emerging as a model species for genetic and molecular studies. We report the draft genome sequence of the Indian strain of the type form of *An. stephensi* as a resource and platform for fundamental and translational research. We also provide unique perspectives on *Anopheles* chromosome evolution and offer new insights into mosquito biology and mosquito-parasite interactions.

## Results and discussion

### Draft genome sequence of *An. stephensi*: Assembly and verification

The *An. stephensi* genome was sequenced using 454 GS FLX, Illumina HiSeq, and PacBio RS technologies (Additional file 1: Table S1). The 454 reads comprised 19.4× coverage: 12.2× from single-end reads, 2.2× from 3 kilobase (kb) paired-end reads, 3.4× from 8 kb paired-end reads, and 1.7× from 20 kb paired-end reads. The majority of 454 reads was in the range of 194 to 395 base-pairs (bp) in length. A single lane of Illumina sequencing of male genomic DNA resulted in 86.4× coverage of 101 bp paired-end reads with an average insert size of approximately 200 bp. Ten cells of PacBio RS sequencing of male genomic DNA produced 5.2× coverage with a median length of 1,295 bp. A hybrid assembly combining 454 and Illumina data produced a better overall result than using 454 data alone (Materials and methods). The resulting assembly was further improved by filling gaps with error-corrected PacBio reads and scaffolding with BAC-ends. The current assembly, verified using various methods, contains 23,371 scaffolds spanning 221 Mb. The assembly includes 11.8 Mb (5.3%) of gaps filled with Ns (Table 1), which is slightly lower than the size of gaps in the *An. gambiae* assembly (20.7 Mb, 7.6%). The N50 scaffold size is 1.59 Mb and the longest scaffold is 5.9 Mb. The number of scaffolds is inflated because we choose to set the minimum scaffold length to 500 bp to include repeat-rich short scaffolds. The assembled size of 221 Mb is consistent with the previous estimate of the *An. stephensi* genome size of approximately 235 Mb [21].

**Table 1:** **Assembly statistics**

**Statistic**	**Value**
Scaffolds (n)	23,371
Scaffold N50 size	1,591,355
Maximum scaffold length	5,975,090
Minimum scaffold length	486
Total length of scaffolds	221,309,404
Percent Ns	5.35%
Contigs (n)	31,761
Contig N50 size	36,511
Maximum contig length	475,937
Minimum contig length	347
Total length of contigs	209,483,518
GC percent	44.80%

### Physical mapping

**Figure 1:**
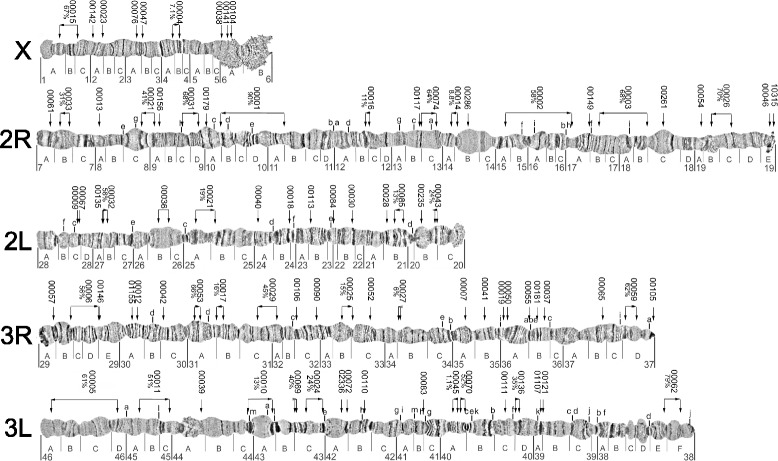
**Physical map.** A physical map of the *An. stephensi* genome was created from FISH on polytene chromosomes comprising 227 probes and 86 scaffolds. These 86 scaffolds comprise 137.14 Mb or 62% of the *An. stephensi* genome. Orientation was assigned to 32 of the 86 scaffolds. The physical map includes 28 of the 30 largest scaffolds.

Mapping of 227 probes was sufficient to assign 86 scaffolds to unique positions on the *An. stephensi* polytene chromosomes (Figure 1; Table 2; Additional file 2: Physical Map Data). These 86 scaffolds comprise 137.14 Mb or 62% of the assembled genome. Our physical map includes 28 of the 30 largest scaffolds and we were able to determine the orientation of 32 of the 86 scaffolds. We expect that relatively little of the heterochromatin was captured in our chromosomal assembly based on the morphology of the chromosomes in regions to which the scaffolds mapped. For this reason, subsequent comparisons with *An. gambiae* on molecular features of the genome landscape exclude regions of known heterochromatin from the *An. gambiae* dataset. *An. stephensi* and *An. gambiae* have different chromosome arm associations with 2L of *An. gambiae* homologous to 3L of *An. stephensi* [22]. Therefore, all ensuing discussion of synteny between the two species refers to *An. stephensi* chromosome arms listed in homologous order to those of *An. gambiae*: X, 2R, 3L, 3R, and 2L. While draft genomes also are available for *An. darlingi* and *An. sinensis* [23,24], we focused our comparative analysis on *An. stephensi* and *An. gambiae*, the only two species that have chromosome-based assembly.

**Table 2:** **Physical map information**

**Arm**	**Scaffolds per arm (n)**	**Length (Mb)**	**Mapped genome (%)**	**Total genome (%)**
X	9	14.95	10.90	6.77
2R	21	39.50	28.80	17.87
2L	15	22.40	16.33	10.14
3R	24	37.83	27.59	17.12
3L	17	22.45	16.37	10.16
Total	86	137.14	100	62.05

Scaffolds mapped to each chromosome, total bp to each chromosome, percent of the predicted genome covered.

### Gene annotation

**Figure 2:**
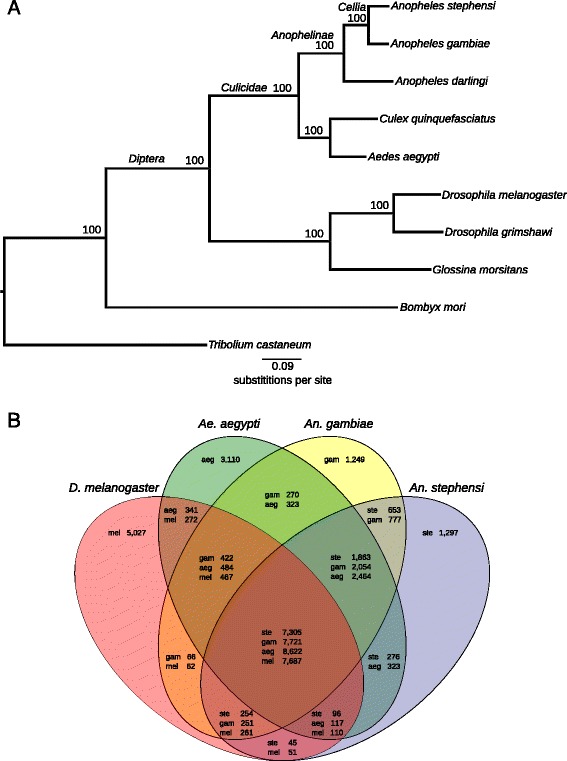
**Molecular species phylogeny and orthology. (A)** The maximum likelihood molecular species phylogeny estimated from universal single-copy orthologs supports the recognized species relationships with *An. stephensi* and *An. gambiae* in subgenus *Cellia* within the genus *Anopheles*. **(B)** Comparative analysis of orthologs from *An. stephensi*, *An. gambiae*, *Ae. aegypti*, and *D. melanogaster*. Orthologous genes were retrieved from OrthoDB. A total of 7,305 genes were shared among all four species, 1,297 genes were specific to *An. stephensi*, 653 genes were *Anopheles*-specific, and 1,863 genes were mosquito-specific.

A total of 11,789 protein-encoding genes were annotated using a combination of homology and *de novo* prediction. These gene models have been submitted to the NCBI (GCA_000300775.2) and are hosted in VectorBase [25]. The average transcript length was 3,666 bp and the average number of exons per transcript was 4.18. Evolutionary relationships among *An. stephensi* and other *dipteran* insects were evaluated by constructing a maximum likelihood molecular species phylogeny using universal single-copy orthologs (Figure 2A). *An. stephensi* and *An. gambiae* form a well-supported clade representing the subgenus *Cellia* within the genus *Anopheles*. This phylogeny provides the evolutionary context for current and future comparative genomics analysis. A total 10,492 (89.0%) of the 11,789 predicted *An. stephensi* protein-encoding genes had orthologs in *An. gambiae*, *Aedes aegypti*, and *Drosophila melanogaster* (Figure 2).

### Global transcriptome analysis

**Figure 3:**
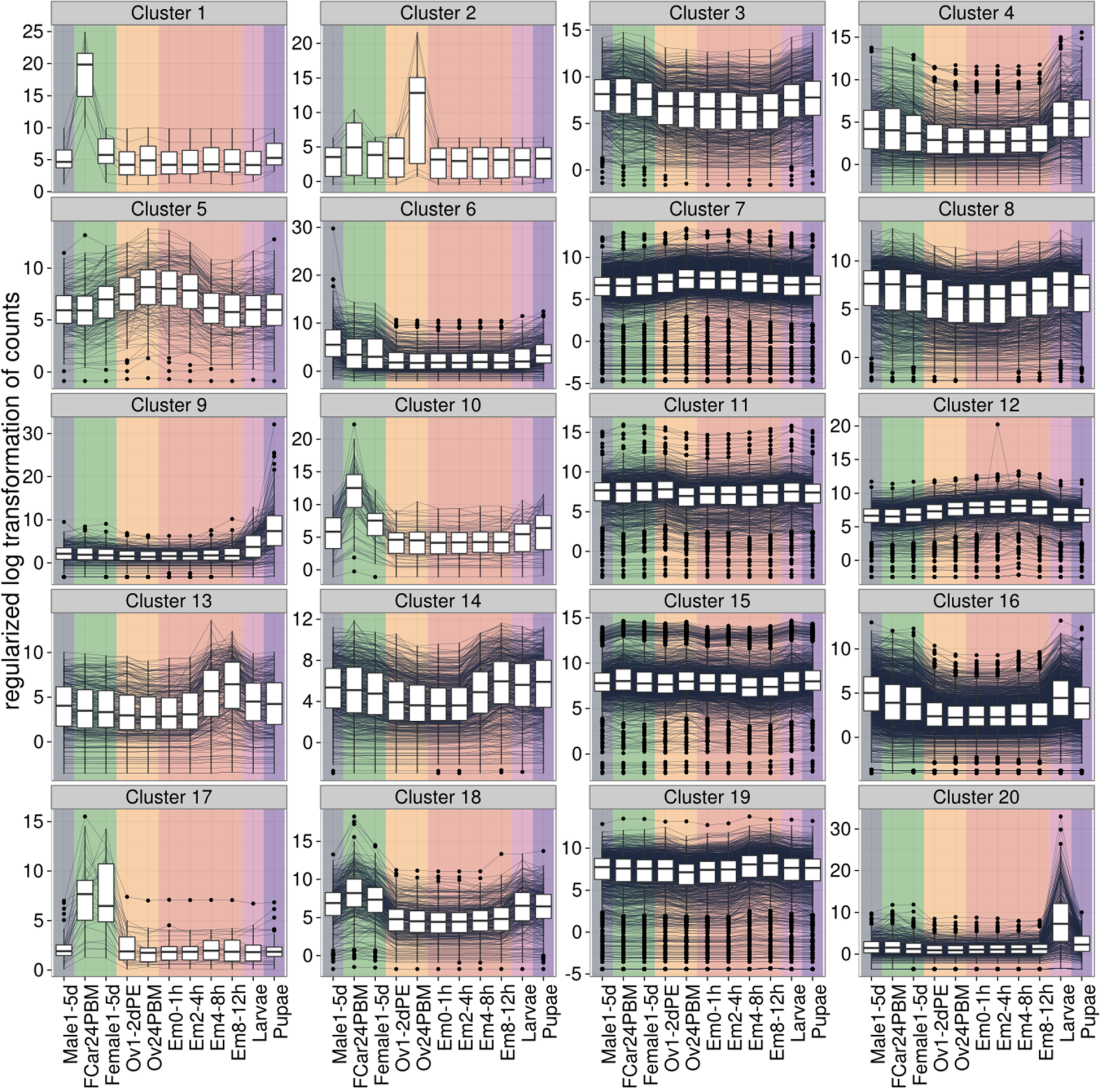
**Gene clustering according to expression profile.** Twenty groups of genes were clustered by expression profile. The expression profiles used for grouping were generated using 11 RNA-seq samples spanning developmental time points including: 0 to 1, 2 to 4, 4 to 8, and 8 to 12 h embryos, larva, pupa, adult males, adult females, non-blood-fed ovaries, blood-fed ovaries, and 24 h post-blood-fed female carcass without ovaries. Male stage are colored blue, female stages are colored green, ovary samples are colored yellow, embryo samples are colored red, larva samples are colored pink, and pupa samples are colored purple. Many of these clusters correspond to either a specific developmental stage or specific sex.

Eleven RNA-seq samples were prepared from 0 to 1, 2 to 4, 4 to 8, and 8 to 12 h post-egg deposition embryos, larvae, pupae, adult males, adult females, non-blood-fed ovaries, blood-fed ovaries, and 24 h post-blood-fed female carcasses without ovaries [26]. The corresponding genes were clustered into 20 distinct groups in sizes in the range of 8 to 2,106 genes per group on the basis of similar expression patterns (Figure 3). Many of the clusters correspond to either a specific developmental stage or sex (Additional file 2). A search for over-represented gene ontology (GO) terms in the 20 clusters found that many of the co-regulated genes have similar inferred functions or roles. Adult females require a protein-rich blood-meal for oogenesis and thus are the most interesting sex from a health perspective. Genes in clusters 1, 10, and 17 are induced in the female soma after blood-feeding. These clusters are enriched for genes encoding proteins with proteolytic activity, including serine peptidases, and involved in blood-meal digestion. Mosquitoes have undergone lineage-specific amplification of serine peptidases when compared to *Drosophila*, many of which are found in the three clusters described above. Cluster 9 contains 258 genes that showed peak expression in the pupal stage and it is enriched for genes whose products are involved in exoskeleton development. GO analyses of other clusters are described in the Additional file 1: Text.

We identified 241 and 313 genes with female- or male-biased expression, respectively (Additional file 2: Sex-biased genes list and GO terms). The male-biased genes are enriched for those whose products are involved in spermatogenesis and the auditory perception. Male mosquitoes detect potential mates using their Johnston’s organ, which has twice the number of sensory neurons as that of the females [27,28]. The female-biased genes are enriched for those whose products are involved in proteolysis and other metabolic processes likely relevant to blood digestion.

### Immunity genes

Manual annotation was performed on genes involved in innate immunity including those that encode the LRR immune (LRIM) and the *Anopheles Plasmodium*-responsive leucine-rich repeat 1 (APL1) proteins, and the genes of the Toll, immune deficiency (IMD), insulin/insulin-like growth factor signalling (IIS), mitogen-activated protein kinase (MAPK), and TGF-β signalling pathways. A number of studies have demonstrated the importance of these genes or pathways in mosquito defense against parasites or viruses [16-20,29-31]. Manual analysis showed overall agreement with the automated annotation and improved the gene models in some cases (Additional file 2). A high level of orthology is generally observed between *An. stephensi* and *An. gambiae* and we highlight here a few potentially interesting exceptions. *An. stephensi* may have only one APL1 gene (ASTEI02571) instead of the three APL1 gene cluster found in *An. gambiae* (Additional file 1: Figure S1)*.* We also observed the apparent lack of TOLL1B and 5B sequences in *An. stephensi*, which in *An. gambiae* are recent duplications of TOLL1A and 5A, respectively.

Expression profiles of all immunity genes were analyzed using the 11 RNA-seq samples to provide insights into their biological functions (Additional file 2: RNA-seq expression profile of immunity-related genes). For example, FKBP12, a protein known to regulate both transforming growth factor (TGF)-β and target of rapamycin (TOR) signaling, showed abundant transcript levels across immature stages and adult tissues (Additional file 1: Figure S2). The high expression levels of AsteFKBP12 in all examined stages and tissues were unexpected. Examination of existing publicly-available microarray data confirmed these expression levels and patterns [32]. FKBP12 in mammals forms a complex with rapamycin and FKBP-rapamycin-associated protein (FRAP) to inhibit TOR [33]. Given that TOR signaling is fundamental to many biological functions in mammals [34] and cumulative data support the same for *D. melanogaster* [35], a high level of FBKP12 expression may be critical for tight regulation of TOR activity in *An. stephensi* and perhaps *An. gambiae* [36]. Expression patterns of the *An. gambiae* FKBP12 ortholog, AGAP012184, from microarray datasets [37] support the hypothesis that this protein is involved in a broad array of *Anopheline* physiologies including: development, blood-feeding, molecular form-specific insecticide resistance, circadian rhythms, desiccation resistance, mating status, and possibly also broad regulation of infection based on studies with murine (*Plasmodium berghei*) and human (*Plasmodium falciparum*) malaria parasites. Whether these same physiologies and others are regulated by FKBP12 in *An. stephensi* will require experimental confirmation. Given that signalling pathways regulating embryonic pattern formation in *Drosophila* (for example, the Toll pathway [38]) have been co-opted in the adult fly for regulation of various physiologies including metabolism and immune defense, the data presented here support the hypothesis that pathways integral to adult biology in adult *Anophelines* also have been similarly co-opted from important developmental roles.

### Salivary genes

Saliva of blood-feeding arthropods contains a cocktail of pharmacologically active components that disarm vertebrate host’s blood clotting and platelet aggregation, induce vasodilation, and affect inflammation and immunity. These salivary proteins are under accelerated evolution due most likely to their host’s immune pressure. A previous salivary gland transcriptome study identified 37 corresponding salivary proteins in *An. stephensi*, most of which are shared with *An. gambiae*, including mosquito and *Anopheles*-specific protein families [39]. A more extensive sialotranscriptome based on approximately 3,000 ESTs identified the templates for 71 putative secreted proteins for *An. gambiae* [40]. The combined data verify the identity of 71 putative salivary secreted proteins for *An. stephensi*, seven of which have no similarities to *An. gambiae* proteins (Additional file 2: Automatic annotated salivary genes). The current assembly of the *An. stephensi* genome shows that many salivary gland genes are present as tandem repeated genes and represent families that arose by gene duplication events. Tandem repeated gene families often are poorly annotated by automated approaches, therefore, manual annotation was necessary to improve the salivary gland gene models (Additional file 2). In particular, *An. gambiae* has eight genes of the D7 family, which has modified odorant binding domains (OBD) that strongly bind agonists of platelet aggregation and vasoconstriction (histamine, serotonin, epinephrine, and norepinephrine) [41]. Three of these genes have two OBDs while the remaining five have only one domain each. As in *An. gambiae*, the short forms are oriented in tandem and in the opposite orientation of the long-form genes. However, *An. stephensi* has apparently collapsed the second long form to create a sixth short form.

### Comparative analysis of additional gene families

Functional annotations of a number of gene families in *An. stephensi* were obtained based on their InterPro ID [42] (Additional file 2: Gene families counts table). We also compared gene numbers in these gene families across several species. *An. stephensi* and *An. gambiae* showed similar gene numbers in most of the gene families [3] and this is consistent with the close phylogenetic relationship between the two species. As observed with manually annotated immunity-related genes (Additional file 1: Figure S3), strong one-to-one relationship was observed between *An. stephensi* and *An. gambiae* genes in odorant binding proteins (OBPs) (Additional file 1: Figure S4A) and other gene families studied. There are a few gene families that showed obvious difference in numbers between *An. stephensi* and *An. gambiae*. We performed phylogenetic analysis of these gene families. The results (Additional file 1: Figure S4B and Figure S4C) indicate gene expansion in the odorant receptors (OR) and fibrinogen-related proteins in *An. gambiae*. Interestingly, a plurality of expanded genes is physically clustered in *An. gambiae*, suggesting that the gene expansions in *An. gambiae* may have arisen from local duplications. For example, the *An. stephensi* single-copy OR gene ASTEI08685 has four orthologs in *An. gambiae* (AGAP004354, AGAP004355, AGAP004356, and AGAP004357). The putative orthologs of these ‘expanded’ genes tend to be single- or low-copy in *An. stephensi* and other related species in Vectorbase, supporting the interpretation that the lack of duplicated copies in *An. stephensi* is not due to assembly or annotation error. Further analysis that includes all species in the ongoing 16 *Anopheles* genomes project [43] will facilitate future comparative analysis of gene family expansions and gene losses.

### Repeat content

Transposable elements (TEs) and other unclassified interspersed repeats constitute 7.1% of the assembled *An. stephensi* genome (Table 3: Additional file 2: Repeat sequences). TE occupancy of the euchromatic genome in *D. melanogaster* and *An. gambiae* is 2% and 16%, respectively [3]. Thus variations in the size of the genomes correlate with different amounts of repetitive DNA in these three species. More than 200 TEs have been annotated. DNA transposons and miniature inverted-repeat TEs (MITEs) comprise 0.44% of the genome. Non-LTR retrotransposons (or LINEs) comprise 2.36% of the genome. Short intersperse nuclear elements (SINEs), although less than 300 bp in length, are highly repetitive and comprise 1.7% of the genome. There is considerable diversity among the LTR-retrotransposons although they occupy only 0.7% of the genome. Approximately 2% of the genome consists of interspersed repeats that remain to be classified.

**Table 3:** **Transposable elements and other interspersed repeats**

**Type**	**Elements (n)**	**Length occupied (bp)**	**Genome (%)**
SINEs	30,514	3,739,253	1.69
LINEs	22,022	5,231,240	2.36
LTR elements	4,359	1,499,282	0.68
DNA elements	4,611	966,667	0.44
Unclassified	30,611	4,322,468	1.95
Total	92,117	15,758,910	7.12

### Genome landscape: a chromosomal arm perspective

**Figure 4:**
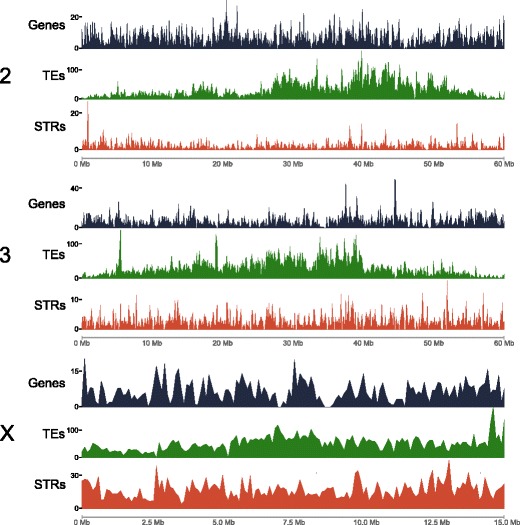
**Genome landscape.** Density of genes (black vertical lines), transposable elements (TEs; green vertical lines), and short tandem repeats (STRs; red vertical lines) in 100 kb windows of mapped scaffolds. Based on the physical map, scaffolds were ordered and oriented respective to their position in the chromosomes and then 100 kb non-overlapping windows were generated for each scaffold (X-axis). The density of genes and TEs (Y-axis) was determined using coverageBed. Satellite sequences were identified using TandemRepeatFinder. The short tandem repeats track is a combination of the number of microsatellites, minisatellites, and satellites per 100 kb window.

**Figure 5:**
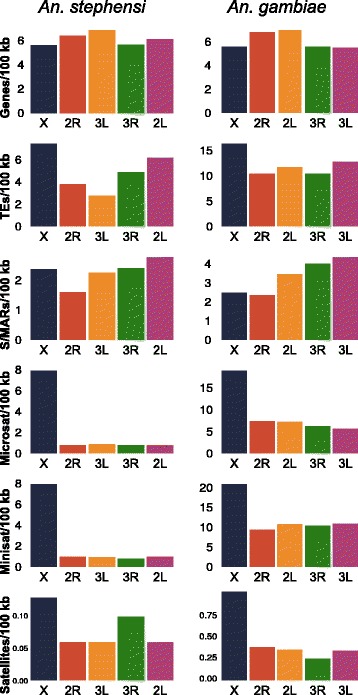
**Average density/100 kb/ARM.** A comparison of the average density per 100 kb of genes, TEs, S/MARS, microsatellites, minisatellites, and satellites between chromosome arms.

The density of genes, TEs, and short tandem repeats (STRs) for each chromosome were determined based on the physical map (Figure 4). The average numbers of genes for each chromosome arm are consistent with those in *An. gambiae*. The X had the lowest number of genes per 100 kb, and the highest densities of genes per 100 kb were seen on 2R and 3 L (Figure 5; Additional file 1: Tables S2 and S3). Chromosomes 2R and 3 L also contain the greatest numbers of polymorphic inversions [44]. Genes functioning as drivers of adaptation could be expected to occur in greater densities on chromosome arms with higher numbers of polymorphic inversions [45].

*An. stephensi* has a lower density of transposable elements across all chromosome arms than *An. gambiae* (Figure 5; Additional file 1: Tables S2 and S3; Additional file 2: Genome Landscape). The density of transposable elements on the *An. stephensi* X is more than twice that of the autosomes. A comparison of the *An. stephensi* simple repeats with those in *An. gambiae* euchromatin showed that densities in the latter were approximately 2-2.5× higher (Figure 5; Additional file 1: Tables S2 and S3). The greatest densities of simple repeats were found on the X chromosome and this is consistent with a previous study in *An. gambiae* [46]. Although *An. stephensi* shows lower densities of simple repeats across all arms compared to *An. gambiae*, its X appears to harbor an over-representation of simple repeats compared to its autosomes. Scaffold/Matrix-associated regions (S/MARs) can potentially affect chromosome mobility in the cell nucleus and rearrangements during evolution [47,48] and these were found to be enriched in the 2 L and 3R arms (Figure 5; Additional file 1: Tables S2 and S3).

### Molecular organization of pericentric heterochromatin

**Figure 6:**
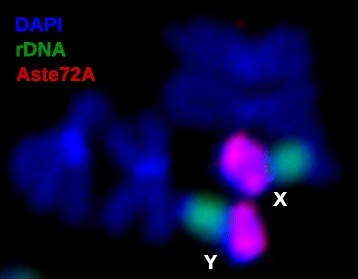
**FISH with Aste72A, rDNA, and DAPI on mitotic chromosomes.** The pattern of hybridization for satellite DNA Aste72A on mitotic sex chromosomes of *An. stephensi*. Aste72A hybridizes to pericentric heterochromatin in both X and Y chromosomes while ribosomal DNA locus maps next to the heterochromatin band in sex chromosomes.

We observed clear differences in heterochromatin staining patterns when comparing mitotic chromosome squashes prepared from imaginal discs of *An. gambiae* and *An. stephensi. An. stephensi* appears to have more pericentric heterochromatin than *An. gambiae* (Additional file 1: Figure S5)*.* This is particularly evident in the sex chromosomes. Mitotic X chromosomes in *An. stephensi* possess much more pericentric heterochromatin compared with X chromosomes from several different strains of *An. gambiae*. Finally, the Y chromosome in *An. stephensi* has a large block of heterochromatin. We further investigated whether particular tandem repeats are concentrated in heterochromatin. Aste72A and Aste190A, the two repeats with highest coverage in raw genomic data reads, were selected as probes for FISH analysis (Additional file 2: Tandem repeat sequences). Aste72A, which comprises approximately 1% of the raw genomic reads, was mapped to the pericentric heterochromatin of X and Y chromosomes (Figure 6). Aste190A, which comprises approximately 2% of the raw genomic reads, was mapped to centromere of both autosomes (Additional file 1: Figure S6). The Aste72A tandem repeat has a 26.7% mean GC content and contributes significantly to the AT-rich peak in the plot of GC distribution of raw genomic reads (Additional file 1: Figure S7).

### Y chromosome

*Anopheles* mosquitoes have heteromorphic sex-chromosomes where males are heterogametic (XY) and females homogametic (XX) [49]. The high repetitive DNA content of Y chromosomes makes them difficult to assemble and they often are ignored in genome projects. An approach called the chromosome quotient [50] was used to identify 57 putative Y sequences spanning 50,375 bp (Additional file 2). All of these sequences are less than 4,000 bp in length and appear to be highly repetitive. Five BACs that appeared to be Y-linked based on the CQs of their end sequences were analyzed by sequencing and their raw PacBio reads were assembled with the HGAP assembler [51]. Eleven contigs spanning 196,498 bp of predicted Y-linked sequences were obtained (Additional file 2). The 57 Y-linked sequences and 11 contigs from the Y-linked BACs represent currently the most abundant set of Y sequences in any *Anopheles* species. RepeatMasker analysis using the annotated *An. stephensi* interspersed repeats showed that approximately 65% of the *An. stephensi* Y sequences are interspersed repeats. LTR retrotansposons alone occupy approximately 49% of the annotated Y (Additional file 2).

### Synteny and gene order evolution

**Figure 7:**
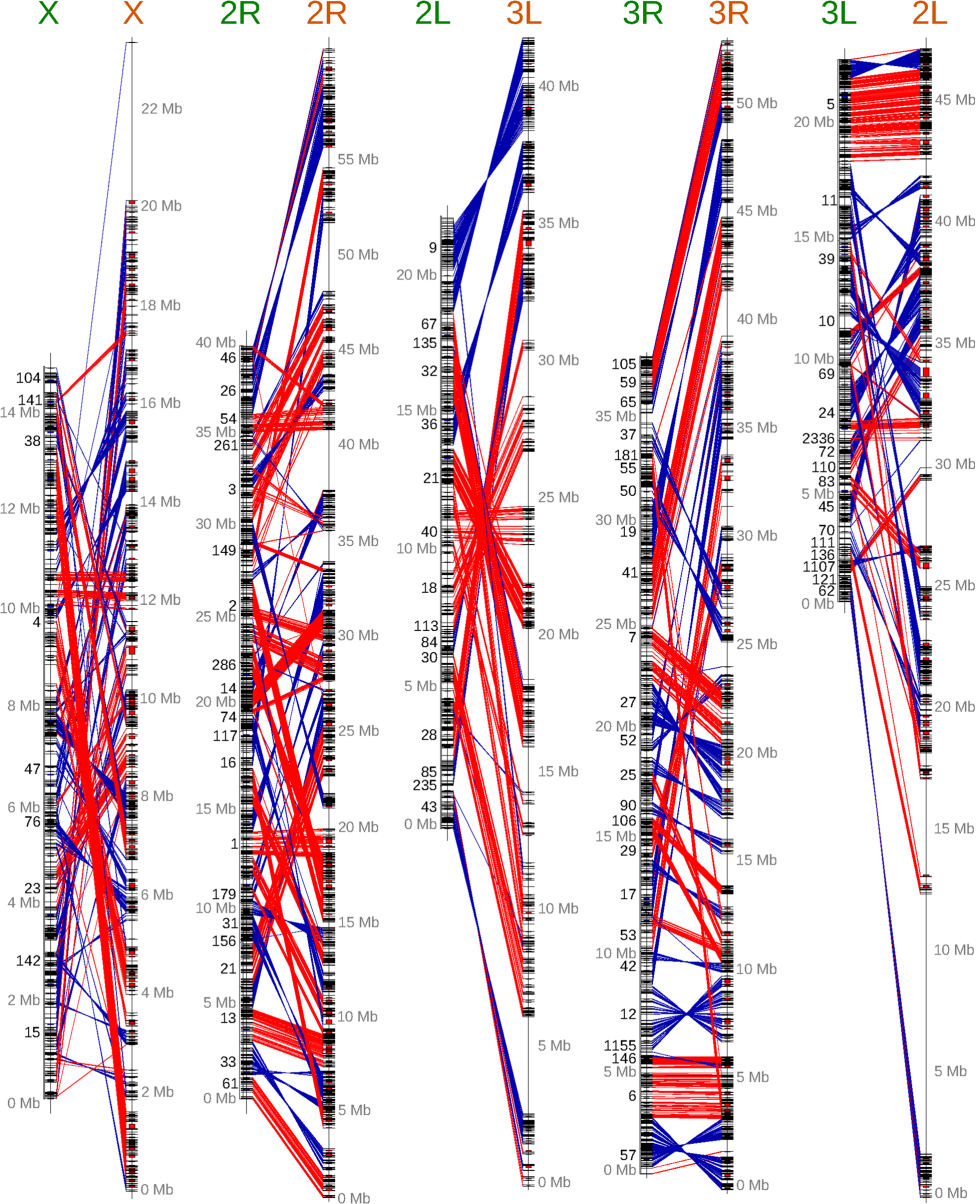
**Synteny.** Synteny between *An. stephensi* and *An. gambiae* based on 6,448 single-copy orthologs. Orthologs with the same orientation in *An. stephensi* and *An. gambiae* are connected with red lines and orthologs with the opposite orientation are connected with blue lines. Orthologous genes from *An. stephensi* and *An. gambiae* were retrieved from OrthoDB. The physical map was used to identify the relative locations of genes on the *An. stephensi* chromosomes. The relationship of the position between the *An. stephensi* and *An. gambiae* orthologs were plotted with GenoPlotR. 66 syntenic blocks were identified on the X chromosome. A total of 104 and 64 syntenic blocks were identified on 2R and 2L (3L in *An. stephensi*). A total of 104 and 42 syntenic blocks were identified on 3R and 3L (2L in *An. stephensi*). Therefore, the X chromosome has undergone the most rearrangements per megabase.

**Figure 8:**
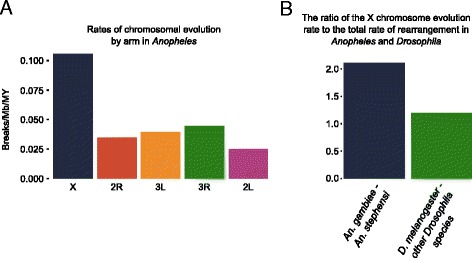
**Chromosome evolution in** ***Anopheles*** **and** ***Drosophila*** **. (A)** Higher rates of rearrangement on the X chromosome compared to autosomes between *An. stephensi* and *An. gambiae*. Arm designations for the figure are according to *An. stephensi*. **(B)** The ratio of the X chromosome evolution rate to the total rate of rearrangement is higher in *Anopheles* than in *Drosophila*.

We used the chromosomal location and orientation of 6,448 one-to-one orthologs from *An. gambiae* and *An. stephensi* to examine synteny and estimate the number of chromosomal inversions between these two species (Figure 7; Additional file 2: Synteny Blocks). Syntenic blocks were defined as those that had at least two genes and all genes within the block had the same order and orientation with respect to one another in both genomes. The X chromosome has markedly more inversions than the autosomes. The number of chromosomal inversions that might have happened since *An. stephensi* and *An. gambiae* last shared a common ancestor was determined with GRIMM [52]. We calculated the density of inversions per chromosome arm ignoring breakpoint reuse and assuming two breakpoints per inversion (Additional file 1: Tables S4 and S5). The length of *An. stephensi* assembly was used as a proxy for the size of the *An. stephensi* chromosomes. The density of inversions per megabase on the X chromosome supports the conclusion that it is much more prone to rearrangement than the autosomes. Genomic segments on the X are approximately three-fold more likely to change order than those on the autosomes (Figure 8A and Additional file 1: Table S6). The fast rate of X chromosome rearrangements contrasts with the lack of polymorphic inversions in *An. stephensi* and *An. gambiae* (Additional file 1: Table S5). Interestingly, a recent comparative genomic study between *An. gambiae* and *Ae. aegypti* revealed that the homomorphic sex-determining chromosome in *Ae. aegypti* has a higher rate of genome rearrangements than autosomes [53].

### Rates of chromosome evolution in *Drosophila* and *Anopheles*

Recent studies have established that both *Anopheles* and *Drosophila* species have high rates of chromosomal evolution as compared with mammalian species [46,54-61]. We compared the number of breaks per megabase for the X chromosome and all chromosomes to understand the differences in the dynamics of chromosome evolution between *Drosophila* and *Anopheles* (Additional file 1: Table S7). These results reveal a higher ratio of the rates of evolution of sex chromosome to all chromosomes in *Anopheles* than *Drosophila*, with means of 2.116 and 1.197, respectively (Figure 8B). We correlated densities of different molecular features including simple repeats, TEs, genes, and S/MARs with the rates of rearrangement calculated for each arm (Additional file 1: Tables S8-S13). The strongest correlations were found among the rates of evolution across all chromosome arms and the densities of microsatellites, minisatellites, and satellites in both *An. gambiae* and *An. stephensi*. The highly-positive correlations between rates of inversion across all chromosome arms and satellites of different sizes are due most likely to the co-occurring abundance of satellites and inversions on the X chromosome. Rates of inversions and satellite densities are much lower on the autosomes. S/MARs in autosomes were correlated negatively and genes correlated positively with polymorphic inversions.

### Genetic diversity of the genome

The genome sequencing effort reported in the current study is based on an inbred laboratory strain to ensure good assembly. Nonetheless, we performed genome-wide SNP analysis based on the available data. A total of 530,997 SNPs were detected (Additional file 2: SNP analysis raw data). A total of 319,751 SNPs were assigned to chromosomes based on mapping information (Additional file 1: Table S14). The SNP calls were assessed for their effect on the primary sequence of transcripts (Additional file 2: Summary of transcript consequences for *An stephensi* Indian strain SNP calls). These analyses will help future population genomic studies and facilitate association studies. We found that the X chromosome has a markedly lower frequency of SNPs than the autosomes in agreement with the similar observation in *An. gambiae* [3]. The observed pattern may be explained by a smaller effective population size of the X chromosome due to male hemizygosity and lower sequence coverage of the X chromosome [62].

## Conclusions

The genome assembly of the type-form of the Indian strain of *An. stephensi* was produced using a combination of 454, Illumina, and PacBio sequencing and verified by analysis of BAC clones and ESTs. Physical mapping was in complete agreement with the genome assembly and resulted in a chromosome-based assembly that includes 62% of the genome. Such an assembly enabled analysis of chromosome arm-specific differences that are seldom feasible in next-gen genome projects.

Comparative analyses between *An. stephensi* and *An. gambiae* showed that the *Anopheles* X has a high rate of chromosomal rearrangement when compared with autosomes, despite the lack of polymorphic inversions in the X chromosomes in both species. Additionally, the difference between the rates of X chromosome and all chromosome evolution is much more striking in *Anopheles* than in *Drosophila.* The high rate of evolution on the X correlates well with the density of simple repeats. Our data indicate that overall high rates of chromosomal evolution are not restricted to *Drosophila* but may be a feature common to *Diptera*.

The genome landscape of *An. stephensi* is characterized by relatively low repeat content compared to *An. gambiae. An. stephensi* appears to have larger amount of repeat-rich heterochromatin in pericentric regions but far less repetitive sequences in chromosomal arms as compared with *An. gambiae*. Using a newly developed chromosome quotient method, we identified a number of Y-chromosome contigs and BACs, which together represent currently the most abundant set of Y sequences in any *Anopheles* species.

The current assembly contains 11,789 predicted protein coding genes, 127 miRNA genes, 434 tRNA genes, and 53 fragments of rRNA genes. *An. stephensi* appears to have fewer gene duplications than *An. gambiae* according to orthology analysis, which may explain the slightly lower number of gene models.

This genome project is accompanied by the first comprehensive RNA-seq-based transcriptomic analysis of an *Anopheles* mosquito. Twenty gene clusters were identified according to gene expression profiles, many of which are stage- or sex-specific. GO term analysis of these gene clusters provided biological insights and leads for important research. For example, male-biased genes were enriched for genes involved in spermatogenesis and the auditory perception.

Close attention was paid to genes involved in innate immunity including LRIMs, APL1, and proteins in the Toll, IMD, insulin, and TGF-β signaling pathways. A high level of orthology is generally observed between *An. stephensi* and *An. gambiae*. RNA-seq analysis, which was corroborated by other expression analysis methods, provided novel insights. For example, a protein known to interact with both TOR and TGF-β signaling pathways showed abundant mRNA expression in a wide range of tissues, providing new leads for insights into both TOR and TGF-β signaling in mosquitoes*.*

## Material and methods

### Strain selection

The Indian strain of *An. stephensi*, a representative of the type form was sequenced. The lab colony from which we selected mosquitoes for sequencing was originally established from wild mosquitoes collected in India. The lab colony has been maintained continuously for many generations so we did not attempt to inbreed it.

### Sample collection

DNA was isolated from more than 50 adult male and female *An. stephensi* using the Qiagen (Hilden, Germany) DNeasy Blood and tissue kit following the suggested protocol. The integrity of the DNA was verified by running an aliquot on a 1% agarose gel to visualize any degradation. Total RNA was isolated using the standard protocol of the mirVana RNA isolation kit (Life Technologies, Carlsbad, CA, USA) and quality was verified using Bioanalyzer (Agilent Technologies, Santa Clara, CA, USA).

### Sequencing

The *An. stephensi* genome was sequenced to 19.4× coverage using 454 FLX Titanium sequencing performed by the Virginia Bioinformatics Institute (VBI) core laboratory. Sequencing was performed on four different libraries: a single-end shotgun library, and 3 kb, 8 kb, and 20 kb mate-pair libraries. A 200 bp insert size library produced from male *An. stephensi* genomic DNA was prepared and subjected to a single lane of Illumina HiSeq. Genomic DNA from male *An.* sequence was subjected to 10 SMRT cells of Pacific Biosciences (PacBio) v1 sequencing. Only males were sequenced with PacBio because we are interested in increasing the probability of finding Y chromosome sequences. Sanger sequencing performed by Amplicon Express was used to sequence 7,263 BAC-ends.

### Genome assembly

We used several approaches to combine the Illumina and 454 data to generate a better assembly. Newbler can take raw Illumina data as input, so we tried a Newbler assembly with the 454 and Illumina data. However, this resulted in a worse assembly than 454 alone. We had much more success with the strategy used to assemble the *Solenopsis invicta* genome [63]. We assembled the Illumina data first, and then cut the assembly into pseudo-454 reads. These reads were then used along with the real 454 data as input to Newbler [64].

### *De novo* Illumina assembly with Celera

We assembled the paired-end Illumina reads using the Celera assembler [65] with the parameters: ‘overlapper = ovl; unitigger = bogart; utgBubblePopping = 1; kickOutNonOvlContigs = 1; cgwDemoteRBP = 0; cgwMergeMissingThreshold = 0.5; merSize = 14’. The Celera assembler output comprised 41,213 contigs spanning 212.8 Mb. The N50 contig size of this assembly was 16.8 kb.

### *De novo* 454 and Illumina pseudo-454 reads assembly with Newbler 2.8

The contigs of the aforementioned Illumina assembly were shredded informatically into 400 bp pieces with overlapping 200 bp to approximate 454 reads. To artificially simulate coverage depth, we started the shredding at offsets with the values of 0, 10, and 20. Shredding the Illumina assembly resulted in 2,452,038 pseudo-454 reads simulating 4.17× coverage.

We generated an assembly of the 454 and pseudo-454 reads with Newbler 2.8 using the ‘-het -scaffold -large -s 500’ parameters. The resulting assembly contained 23,595 scaffolds spanned 221 Mb. The scaffold N50 size was 1.34 Mb. Mitochondrial DNA (1 scaffold), and other contamination (87 scaffolds) were identified by blastn and removed from the assembly.

### Gap-filling with PacBio reads

PacBio data was used to fill gaps in the scaffolds to further improve the genome assembly. We error-corrected raw PacBio reads using the 454 sequencing data with the Celera pacBioToCa pipeline. pacBioToCa produced 0.88 Gb of error-corrected PacBio reads. Using the error-corrected PacBio data as input, Pbjelly [66] was used to fill gaps with parameters: ‘-minMatch 30 -minPctIdentity 98 -bestn 10 -n Candidates 5 -maxScore -500 -nproc 36-noSplitSubreads’. Pbjelly filled 1,310 gaps spanning 5.4 Mb.

### Further scaffolding with BAC-ends

The scaffolds of the assembly were improved subsequently through the integration of 3,527 BAC-end pairs (120 kb ± 70 kb) using the Bambus scaffolder [67] (Additional file 2: BAC-ends dbGSS accession numbers). The BAC-end sequences were mapped to the scaffolds using Nucmer [68]. The output files were used to generate the ‘.contig’ format files required for Bambus. In total, 275 links between scaffolds were detected. Of these, 169 were retained as potential valid links, which are links connected by uniquely mapped BAC-ends. Links confirmed by less than two BAC-ends were rejected. A total of 46 links were retained that together connected 22 scaffolds, increasing the N50 scaffold size from 1,378 kb to 1,572 kb.

### Assembly validation

#### CEGMA (Core Eukaryotic Genes)

We used CEGMA [69] to search for the number of core eukaryotic genes to test the completeness and correctness of the genome assembly. CEGMA provides additional information as to whether the entire core eukaryotic genes are present (>70%) or only partially present (>20% and <70%). In total, CEGMA found 96.37% of the 248 core eukaryotic genes to be present, and 97.89% of the core eukaryotic genes to be partially present.

#### BAC-ends

We checked whether BAC-ends align concordantly to the genome to study the structural correctness of the *de novo* assembly. BAC-ends were aligned to the scaffolds using NUCMER. In order to ensure unambiguous mapping, only sequences that aligned to a unique location with >95% coverage and 99% identity were used. In total, 21.6% of the BAC-end sequence pairs could be aligned to a unique position in the *An. stephensi* genome with these stringent criteria. Pairs of BAC-end sequence that aligned discordantly to a single scaffold were considered indicative of potential misassembly. Only four of 717 aligned BAC-end pairs aligned discordantly with the assembly confirming overall structural correctness.

#### ESTs

*An. stephensi* EST sequences were downloaded from both the NCBI and VectorBase. We screened the EST sequences to remove any residual vector sequence. The screened ESTs were aligned to the assembly with GMAP [70]. In total, 35,367 of 36,064 ESTs aligned to the assembly. Of these, 26,638 aligned over at least 95% of their length with an identity of >98%. The high percentage of aligned ESTs demonstrates the near-completeness of the *An. stephensi* genome assembly.

Fluorescent *in situ* hybridization (FISH): Slides were prepared from ovaries of lab reared, half-gravid females of the *An. stephensi* Indian wild-type strain. Slide preparation and hybridization experiments followed the techniques described in Sharakhova *et al.* [71]. Fluorescent microscope images were converted to black and white and inverted in Adobe Photoshop. FISH signals were mapped to specific bands or interbands on the physical map for *An. stephensi* presented by Sharakhova *et al.* [72].

### Constructing the physical map

For the chromosomal based genome assembly, all probes mapped by *in situ* hybridization by Sharakhova [72] and this study were aligned to the final version of the *An. stephensi* genome using NCBI blast + blastn. Different blastn parameters were used for probes from different sources to determine if the probe was kept in the final assembly. An e-value of 1e-40 and an identity of >95% was required for probes from *An. stephensi*. An e-value of 1e-5 was required for probes from species other than *An. stephensi*. Probes that mapped to more than one location in the genome were discarded. The work by Sharakhova *et al.* [72] hybridized 345 probes however, only approximately 200 probes from that study were maintained in the final chromosomal assembly. An additional 27 PCR products and BAC clones were hybridized to increase the coverage of our chromosomal assembly.

### Annotation

The genome assembly was annotated initially using the MAKER pipeline [73]. This software synthesizes the results from *ab initio* gene prediction with experimental gene evidence to produce final annotations. Within the MAKER framework, RepeatMasker [74] was used to mask low-complexity genomic sequence based on the repeat library from previous prediction. First, ESTs and proteins were aligned to the genome by MAKER using BLASTn and BLASTx, respectively. MAKER uses the program Exonerate to polish BLAST hits. Next, within the MAKER framework, SNAP [75] and AUGUSTUS [76] were run to produce *ab initio* gene predictions based on the initial training data. SNAP and AUGUSTUS were run once again inside of MAKER using the initial training obtained from the ESTs and protein alignments to produce the final annotations.

### Orthology and molecular species phylogeny

Orthologs of predicted *An. stephensi* genes were assigned by OrthoDB [77]. Information about orthologous genes for *An. gambiae*, *Ae. aegypti*, and *D. melanogaster* also were downloaded from OrthoDB. Enrichment analysis was performed for categories of orthologs using the methods provided in the ontology section. The molecular phylogeny of the 10 selected species was determined from the concatenated protein sequence alignments using MUSCLE [78] (default parameters) followed by alignment trimming with trimAl [79] (automated1 parameters) of 3,695 relaxed single-copy orthologs (a maximum of three paralogs allowed in no more than two species, longest protein selected) from OrthoDB [77]. The resulting 2,246,060 amino acid columns with 932,504 distinct alignment patterns was analyzed with RAxML [80] with the PROTGAMMAJTT model to estimate the maximum likelihood species phylogeny with 100 bootstrap samples.

### Transcriptomics

RNA-seq from 11 samples including: 0 to 1, 2 to 4, 4 to 8, and 8 to 12 h embryos, larva, pupa, adult males, adult females, non-blood-fed ovaries, blood-fed ovaries, and female carcasses without ovaries as described [26] were used for transcriptome analysis. These RNA-seq samples are available from the NCBI SRA (SRP013839). Tophat [81] was used to align these RNA-seq reads to the *An. stephensi* genome and HTSeq-count [82] was used to generate an occurrence table for each gene in each sample. The numbers of alignments to each gene in each sample then were clustered using MBCluster.Seq [83], an R package designed to cluster genes by expression profile based on Poisson or Negative-Binomial models. MBCluster.Seq generated 20 clusters. To visualize these results we performed regularized log transformation to the original occurrence tables for all 20 clusters using DESeq2 [84]. The results were plotted using ggplot2 [85].

### Ontology

Gene ontology (GO) terms were assigned for the 20 clusters of predicted *An. stephensi* genes. GO terms were assigned using Blast2Go [86]. The predicted proteins are blasted against the NCBI non-redundant protein database and scanned with InterProScan [87] against InterPro’s signatures. After GO terms were assigned, GO-slim results were generated for the available annotation based on the Generic GO slim mapping. The GO terms assigned by Blast2GO were subject to GO term enrichment. Over-represented GO terms were identified using a hypergeometric test using the GOstats package in R [88].

### Functional annotation of key gene families

We obtained the InterPro ID information for proteins in *An. stephensi* from the ontology analysis. We functionally annotated gene families based on the assigned InterPro ID. The gene families, including genes involved in immunity, chemosensation, and detoxification were studied. For comparative genome analysis, we retrieved the InterPro ID for seven other species (*An. gambiae*, *An. darlingi*, *A. aegypti*, *Culex quinquefasciatus*, *D. melanogaster*, *Bombyx mori*, and *Tribolium castaneum*) using Biomart [89] from vectorbase [90] and Ensembl Metazoa [91]. We compared gene numbers in gene families of interest. For gene families with obvious differences in numbers between *An. stephensi* and *An. gambiae*, we preformed phylogenetic analysis of these genes. First we aligned these genes from *Anopheles* species using MUSCLE [78]. Then, we constructed phylogenetic tree using Neighbor-joining method with 1,000 bootstrap replicates by CLC Genomics Workbench 4 [92].

### Non-coding RNA

We used tRNAScan-SE [93] with the default eukaryotic mode to predict 434 tRNAs in the *An. stephensi* genome (Additional file 1: Table S15; Additional file 2: Non-coding RNA annotation). Other non-coding RNAs were predicted with INFERNAL [94] by searching against Rfam database version 11.0 [95]. A total of 53 fragmental ribosomal RNA, 34 snRNA, 7 snoRNA, 127 miRNA, and 148 sequences with homology to the *An. gambiae* self-cleaving riboswitch were predicted with an e-value cutoff of 1e-5.

### Transposable elements and other interspersed repeats

Transposable element discovery and classification were performed on the *An. stephensi* scaffold sequences using previously-described pipelines for LTR-retrotransposons, non-LTR-retrotransposons, SINEs, DNA-transposons, and MITEs, followed by manual inspection [96]. The manually-annotated TE libraries then were compared with the RepeatModeler output to remove redundancy and to correct mis-classification by RepeatModler. A repeat library was produced that contains all manually-annotated TEs and non-redundant sequences from RepeatModeler. The repeat library was used to run RepeatMasker at default settings on the *An. stephensi* assembly to calculate TE copy number and genome occupancy.

### Simple repeats

The number of microsatellites, minisatellites, and satellites present in the mapped scaffolds for each chromosome were derived by dividing the scaffolds into strings of 100,000 bp and then concatenating them into a multi-FASTA file to represent an *An. stephensi* pseudo chromosome. Scaffolds were oriented when possible, and all unoriented scaffolds were given the default positive orientation for that chromosome. The multiFASTA file for each pseudo-chromosome was analyzed using a local copy of TandemRepeatsFinder v 4.07b [97]. Parameters for the analysis followed those used by Xia *et al.* [46]: microsatellites were those of period size 2 to 6 with copy number of >8. Minisatellites had period size 7 to 99 while repeats were considered satellites if they had a period size of >100. Both satellites and minisatellites were considered only if they had a copy number of >2. Simple repeats were recorded only if they had at least 80% identity.

### Identification of S/MARs

Scaffold/matrix associated regions were identified using the SMARTest bioinformatic tool provided by Genomatix [98]. Densities of genes and TEs per 100 kb window were calculated using Bedtools coverage based on the genome annotation and TE annotation, respectively.

### Synteny, gene order evolution, and inversions

One-to-one orthologs from *An. gambiae* and *An. stephensi* were identified using OrthoDB [77] and their locations on the *An. gambiae* and *An. stephensi* scaffolds determined. Comparative positions of the genes on the scaffolds based on ontology relationships were plotted using genoPlotR [99]. Scaffolds that mapped using two or more probes were oriented properly, but those anchored by only one probe were used in their default orientation. The number of synteny blocks for each pair of homologous chromosome arms between *An. stephensi* and *An. gambiae* was determined from the images output from genoPlotR. Two criteria were imposed to determine the number of synteny blocks: the orientation of two or more orthologous genes, and whether the genes remained in the same order on the chromosome of *An. stephensi* as in *An. gambiae*. Thus, a group of two or more genes is assigned to the same synteny block if it has the same orientation and order in both species. Synteny blocks were numbered 1, 2, 3, 4, and so on along the chromosome by assigning *An. gambiae* as the default gene order. *An. stephensi* was considered rearranged compared to *An. gambiae* when the numbering of synteny blocks was the same in both species but the order was rearranged in *An. stephensi*. After quantifying the number of synteny blocks and the amount of gene rearrangement between the two species, we estimated the number of chromosomal inversions between them using the programs Genome Rearrangements in Mouse and Man (GRIMM [52]).

### SNP analysis

We used CLC Genomics Workbench 4 [92] to identify SNPs using a combination of the male and female Illumina data (Accession number: SRP013838). The required coverage was 20 and minimum variant frequency was 35. SNP calls made on the assembly were assessed for their effect on transcripts from the gene build using the Ensembl e-hive, variation database, and variation consequence pipeline (available from github [100] and [101]). The Ensembl variation consequence pipeline uses the Ensembl API in the same manner as the Variant Effect Predictor [102] and produces equivalent output. The variation consequence pipeline directly loaded the analysis results into an Ensembl MySQL variation database which was used to generate summary statistics of transcript consequences classified using Sequence Ontologs [103].

### Data access

The *An. stephensi* genome assembly has been deposited in GenBank under the accession number ALPR00000000 and is available at [90]. The raw sequence data used for genome assembly are available in the NCBI SRA: 454 - SRP037783, Illumina - SRP037783, and PacBio - SRP037783. The BAC-ends used for scaffolding are available from the NCBI dbGSS accession numbers: KG772729 - KG777469. RNA-Seq data can be accessed at the NCBI SRA with ID SRP013839.

## Additional files

Additional file 1: Figure S1. Genome alignments showing possible gene copy number evolution within the APL1 gene family. **Figure S2.** Expression of Aste4e-BP1 and FKBP12 during development and following a bloodmeal relative to a representative subset of other signaling molecules. **Figure S3.** Phylogenetic tree for manually annotated immunity-related genes. **Figure S4.** Phylogenetic tree for *Anopheles* OBPs, OR, and fibrinogen-related proteins. **Figure S5.** Comparison of DAPI stained heterochromatin in mitotic chromosomes between *An. stephensi* and *An. gambiae*. **Figure S6.** FISH with Aste190A, rDNA, and DAPI on mitotic chromosomes. **Figure S7.** The GC content in raw HiSeq reads of *An. stephensi* (left) and *An. gambiae* (right). **Table S1.** Data used for assembly. **Table S2.** *An. stephensi* density/100 kb. **Table S3.** *An. gambiae* density/100 kb. **Table S4.** Synteny blocks and inversions between *An. stephensi* and *An. gambiae.* **Table S5.** Inversion breaks/Mb between *An. stephensi* and *An. gambiae.* **Table S6.** The ratio of the X chromosome evolution rate to the autosomal rate of rearrangement between *An. stephensi* and *An. gambiae*. **Table S7.** The ratio of the X chromosome evolution rate to the total rate of rearrangement in *Anopheles* and *Drosophila*. **Table S8.** Correlation of *An. stephensi* genes and rearrangements*.* **Table S9.** Correlation of *An. gambiae* genes and rearrangements*.* **Table S10.** Correlation of *An. stephensi* S/MARs and rearrangements*.* **Table S11.** Correlation of *An. gambiae* S/MARs and rearrangements*.* **Table S12.** Correlation of *An. stephensi* short tandem repeats and rearrangements*.* **Table S13.** Correlation of *An. gambiae* short tandem repeats and rearrangements*.* **Table S14.** SNPs chromosomal distribution Tables. **Table S15.** tRNA Tables. Additional file 2:
**1.** Physical map data. **2.** Lists of genes in clusters. **3.** Cluster ontology. **4.** Sex-biased genes list and GO terms. **5.** Revised annotation for immunity-related genes. **6.** Sequences of immunity-related genes. **7.** RNA-seq expression profile of immunity-related genes. **8.** Automatic annotated salivary genes. **9.** Revised manual annotation for salivary genes. **10.** Manual annotated salivary genes sequences. **11.** Gene families counts table. **12.** Repeat sequences. **13.** Genome landscape. **14.** Tandem repeat sequences. **15.** Chromosome quotients of putative Y-linked scaffolds. **16.** Sequences of putative Y-linked scaffolds. **17.** Chromosome quotients of Y-linked BACs. **18.** Sequences of Y-linked BACs. **19.** Repeat masker output of Y-linked BACs and Y-linked scaffolds. **20.** Repeat masker output of Y-linked BACs. **21.** Synteny blocks. **22:** SNP analysis raw data. **23.** Summary of transcript consequences for *An. stephensi* Indian strain SNP calls. **24.** BAC-ends dbGSS accession numbers. **25.** Non-coding RNA annotation.
