# Supplementary material for: Genome analysis of a major urban malaria vector mosquito, Anopheles stephensi
Source: Genome Biol. 2014 Sep 23;15(9):459. doi: 10.1186/s13059-014-0459-2 (PMC4195908; doi:10.1186/s13059-014-0459-2)
Supplement: Additional file 1: Figure S1. — Genome alignments showing possible gene copy number evolution within the APL1 gene family. Figure S2. Expression of Aste4e-BP1 and FKBP12 during development and following a bloodmeal relative to a representative subset of other signaling molecules. Figure S3. Phylogenetic tree for manually annotated immunity-related genes. Figure S4. Phylogenetic tree for Anopheles OBPs, OR, and fibrinogen-related proteins. Figure S5. Comparison of DAPI stained heterochromatin in mitotic chromosomes between An. stephensi and An. gambiae. Figure S6. FISH with Aste190A, rDNA, and DAPI on mitotic chromosomes. Figure S7. The GC content in raw HiSeq reads of An. stephensi (left) and An. gambiae (right). Table S1. Data used for assembly. Table S2. An. stephensi density/100 kb. Table S3. An. gambiae density/100 kb. Table S4. Synteny blocks and inversions between An. stephensi and An. gambiae. Table S5. Inversion breaks/Mb between An. stephensi and An. gambiae. Table S6. The ratio of the X chromosome evolution rate to the autosomal rate of rearrangement between An. stephensi and An. gambiae. Table S7. The ratio of the X chromosome evolution rate to the total rate of rearrangement in Anopheles and Drosophila. Table S8. Correlation of An. stephensi genes and rearrangements. Table S9. Correlation of An. gambiae genes and rearrangements. Table S10. Correlation of An. stephensi S/MARs and rearrangements. Table S11. Correlation of An. gambiae S/MARs and rearrangements. Table S12. Correlation of An. stephensi short tandem repeats and rearrangements. Table S13. Correlation of An. gambiae short tandem repeats and rearrangements. Table S14. SNPs chromosomal distribution Tables. Table S15. tRNA Tables. [file 13059_2014_459_MOESM1_ESM.docx]

# Additional file

## GO analysis of orthologous genes

The 7,305 genes shared among all four species (*An. stephensi*, *An. gambiae*, *Aedes aegypti*, *and Drosophila melanogaster*) are enriched for those that encode proteins that play universal roles including: metabolism, translation, RNA processing, and cell cycling. It is not surprising that these genes are well-conserved among the four species. The 1,863 mosquito-specific proteins are highly-enriched for those involved in sensing the environment and proteolysis. The 653 *Anopheles*-specific proteins are enriched for functions involved in sensing the environment and proteolysis. The 1,297 proteins unique to *An. stephensi* are enriched for transcription factors and proteins involved in neural development.

## Global transcriptome dynamics

Cluster 2 contains only 10 genes that are upregulated in the post-bloodmeal ovary when eggs are developing. Most of the genes appear to encode proteins involved in the process of egg development, including those proposed to function in fertilization and egg activation. Interestingly, there also is an odorant binding protein (OBP) upregulated in the post-bloodmeal ovary. Recent studies of mosquito transcriptomes and eggshell proteomes support roles for OBPs in oocyte development [1,2]. Cluster 20 contains 397 genes that are upregulated in the larvae. The majority of these proteins are involved in digestion and this is consistent with the physiology of consumption and growth of the larval stage. Cluster 6 comprises 361 genes that are upregulated first in larvae and continue throughout the remainder of the life stages. This cluster is enriched for genes whose products are involved in sensing the environment, many of which are associated with eye development including opsin and rhodopsin signaling. Cluster 6 also contains a few genes involved in the perception of sound. Interestingly, genes in this cluster are slightly more upregulated in adult males than in adult females. Cluster 16 is a large cluster that contains 1,505 genes highly-expressed in life stages starting with the larva and extending through the remainder of the life stages. It is enriched for genes involved in cell-to-cell communication along with many G-protein coupled receptor pathway genes. It also contains many genes involved in neural development and associated traits including behavior, cognition, and learning.

## Rates of chromosome evolution in *Drosophila* and *Anopheles*

Genome Rearrangements In Mouse and Man (GRIMM) was used to estimate the minimum number of inversions necessary. Syntenic blocks were defined as those that had at least two genes and all genes within the block had the same order and orientation with respect to one another in both genomes. GRIMM reported 47 inversions for X, 42 for 2R, 27 for 3L, 51 for 3R, while only 17 for inversions were estimated for 2L. We used a signed option analysis for GRIMM that considers the direction of the inversion. *Anopheles* and *Drosophila* species are estimated to have diverged approximately 250 MYA and limited microsynteny exists among their chromosomes [3]. Recent studies have established that high rates of chromosomal evolution are common to both groups [4-9]. Synteny comparisons and rates of evolution are available for many more pairs of *Drosophilids* and it was proposed that they possess one of the most malleable eukaryotic genomes. The availability of the physically mapped genomic data for several *Drosophila* species [6] has permitted multispecies comparison of evolutionary rates between genera *Anopheles* and *Drosophila*. The length of the mapped *An. stephensi* assembly and length of the mapped genome assembly for each *Drosophila* species was used as a proxy for the size of chromosomes. A recent multigene phylogeny supports a divergence of approximately 30.4 MYA for *An. stephensi* and *An. gambiae* [10]. A comparison of our *An. stephensi-An. gambiae* evolution rates are similar to those of a number of *Drosophila* pairs [8], with rates being higher in the majority of *Drosophila* species, except in *D. erecta* and *D. yakuba* (Table S7). However, comparison of average rate of evolution for the autosomes *versus* the sex chromosomes reveals a much higher rate of evolution for the *Anopheles* X relative to the autosomes (Table S6). Rates of rearrangements were calculated separately for the X chromosome and for the total mapped genome. We found that the ratio of the rates of evolution of sex chromosome to all chromosomes is higher in *Anopheles* than *Drosophila*, with means of 2.116 and 1.197, respectively. Differences in the rates of evolution of the sex chromosomes relative to the autosomes in these two groups can perhaps be explained by differences in the X chromosomes. In *Drosophila*, Muller’s element A is of comparable length to the autosomes. Previous work in *Anopheles* and *Drosophila* have established that chromosomal arms are evolving at different rates [11,12]. Interestingly, despite the rapid rate of X chromosome rearrangements in *Anopheles*, both *An. stephensi* and *An. gambiae* lack polymorphic inversions on their X chromosomes (Table S5) [13,14]. Several reasons including simple repeats, transposable elements and segmental duplications have been implicated for why some chromosome arms are more prone to breakage and inversions than other arms [11,15-18]. We correlated densities of different molecular features including simple repeats, TEs, genes, and S/MARs with the rates of rearrangement calculated for each arm. Our strongest correlations were found among the rates of evolution across all chromosome arms and the densities of microsatellites, minisatellites, and satellites in both *An. gambiae* and *An. stephensi*. Correlation values in *An. stephensi* were 0.98, 0.97, and 0.90 for micro-, mini-, and satellites, respectively (Table S12). In *An. gambiae* those correlation values were 0.98, 0.94, and 0.94 for micro-, mini-, and satellites, respectively (Table S13). Undoubtedly, the highly-positive correlations between rates of inversion across all chromosome arms and satellites of different sizes are most likely due to the co-occurring abundance of satellites and inversions on the X chromosome. Correlations of rates of inversions on the autosomes to satellites are much lower. From the autosomal perspective, MARs were negatively correlated polymorphic inversions in *An. stephensi* (-0.72) and in *An. gambiae* (-0.80) (Tables S10 and S11). The density of genes was correlated positively with polymorphic inversions in *An. stephensi* (0.87) and *An. gambiae* (0.99) (Tables S8 and S9). This positive correlation is consistent with the interpretation that greater densities of genes would correspond to greater chances that polymorphic inversions could capture genes or groups of genes that confer adaptive advantage.


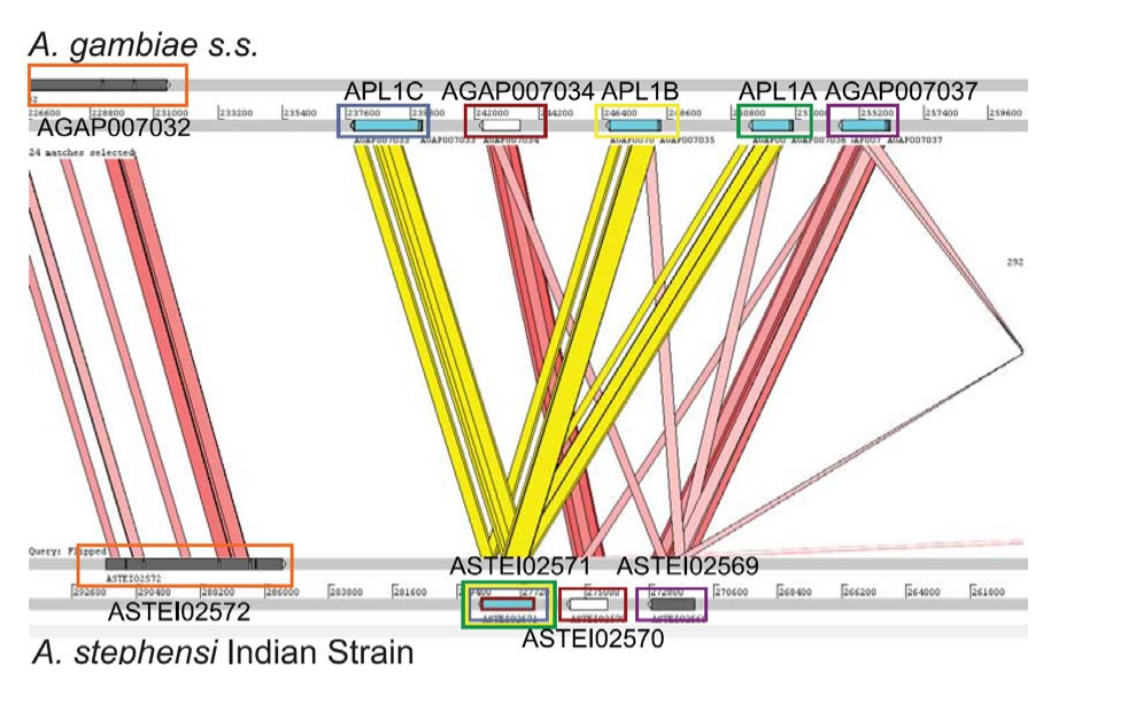


## Figure S1. Genome alignments showing possible gene copy number evolution within the APL1 gene family.

Similarity across genomic regions flanking APL1 is shown using an Artemis Comparison Tool plot [19]. The deeper the shade of red, the greater the similarity across sequences. Yellow color highlights the APL1 gene(s). Flanking genes are conserved between species. It appears that the *An. gambiae* APL1 gene family exists as a single predicted gene within *An. stephensi* (ASTEI02571). Illumina raw data for *An. stephensi*showed no difference in read depth across APL1 and nearby genes, further supporting the hypothesis of a single APL1 gene in *An. stephensi*.


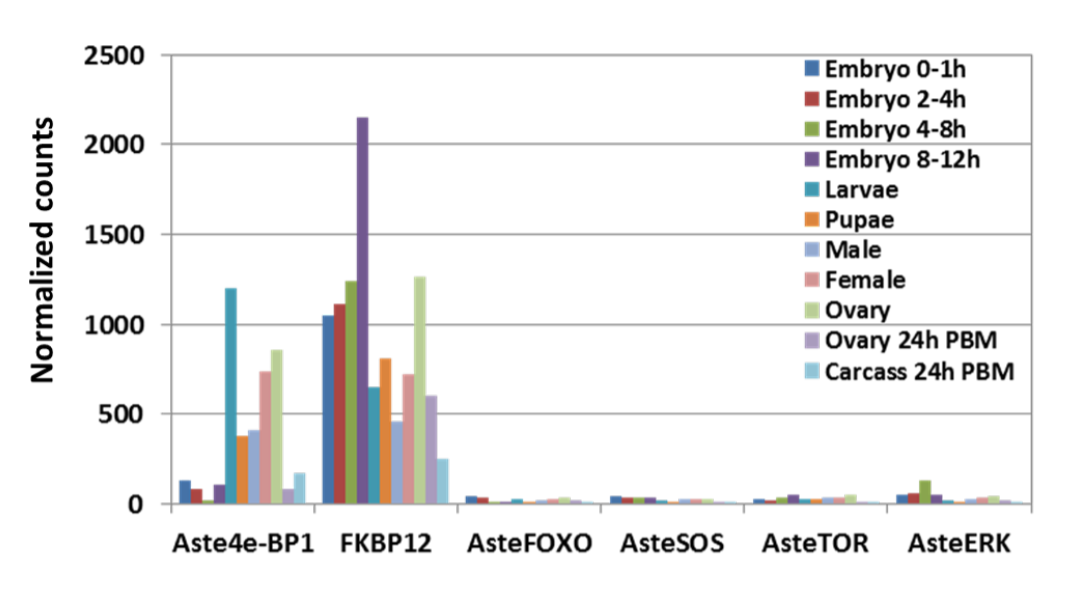


## Figure S2. Expression of Aste4e-BP1 and FKBP12 during development and following a bloodmeal relative to a representative subset of other signaling molecules.

RNA-seq analysis was used to determine the relative transcript expression of 40 IIS, MAPK, TGF-β, and TOR signaling proteins during various developmental stages and adult carcasses and ovaries prior to and 24 h post-bloodmeal (PBM). Of these, Aste4e-BP1 and FKBP12 had dramatically higher expression in nearly all samples. Aste4E-BP1 is a key repressor of translation until phosphorylated by IIS and it was highly expressed in all developmental stages except the embryo. Intriguingly, Aste4E-BP1 expression was reduced in both carcasses and ovaries 24 h after blood-feeding, a period of increased IIS and presumably 4E-BP1 inactivation. Further evaluation at multiple post-bloodmeal timepoints for 4E-BP1 transcript and protein levels as well as phosphorylation status will be necessary to verify RNA-seq results and to understand temporal associations with translation. Analysis of FKBP12 is highlighted in the main text.

A

B

## Figure S3. Phylogenetic tree for manually annotated immunity-related genes.

All trees are NJ trees with 1,000 bootstraps. Some trees are condensed (that is, only branches supported >50% of the time are shown). For leucine-rich repeat immune (LRIM) (A) proteins and Toll-like receptors (TLRs) (B), there is a high level of orthology between *An. stephensi* and *An. gambiae*.

**A**
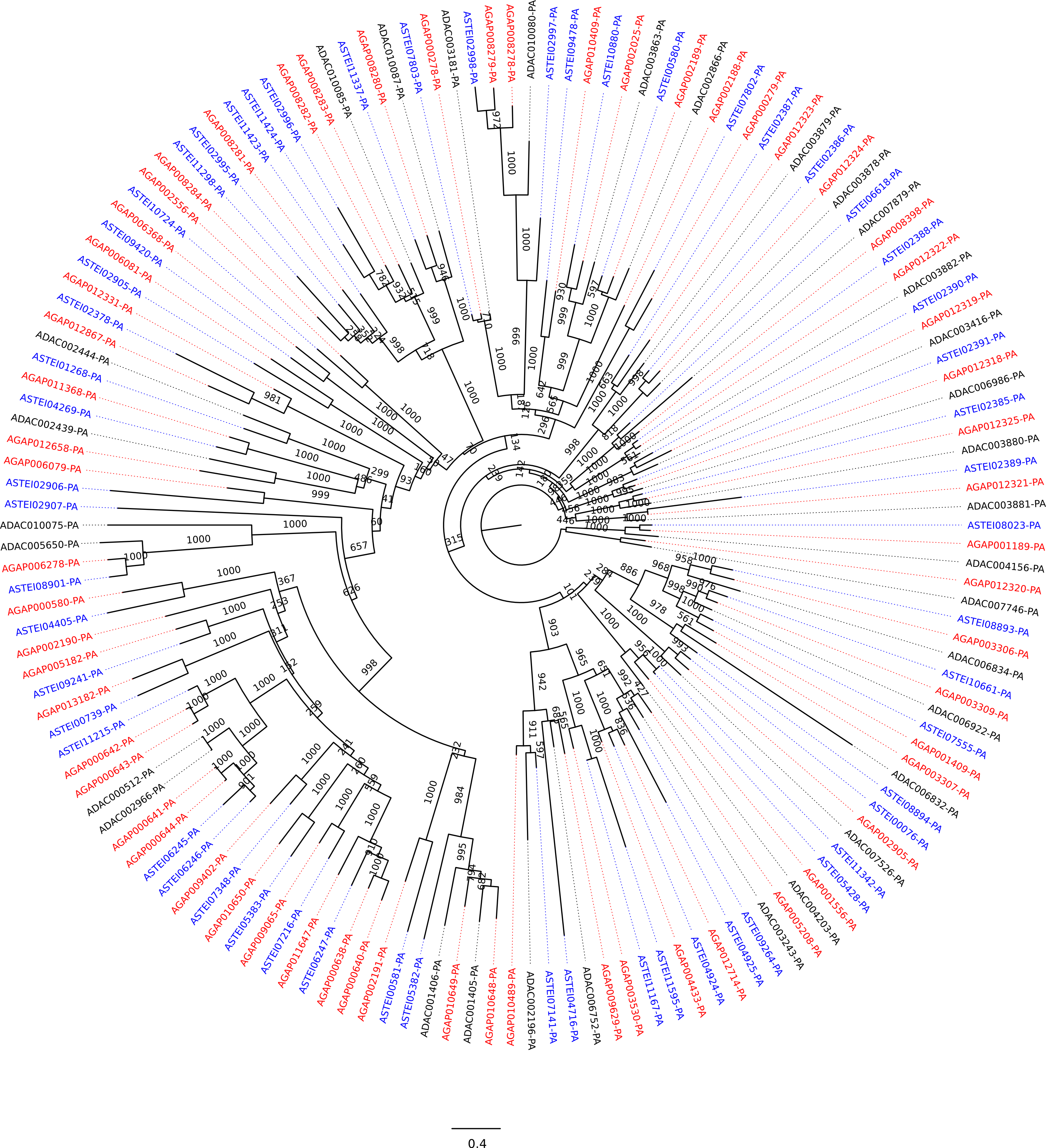


**B**
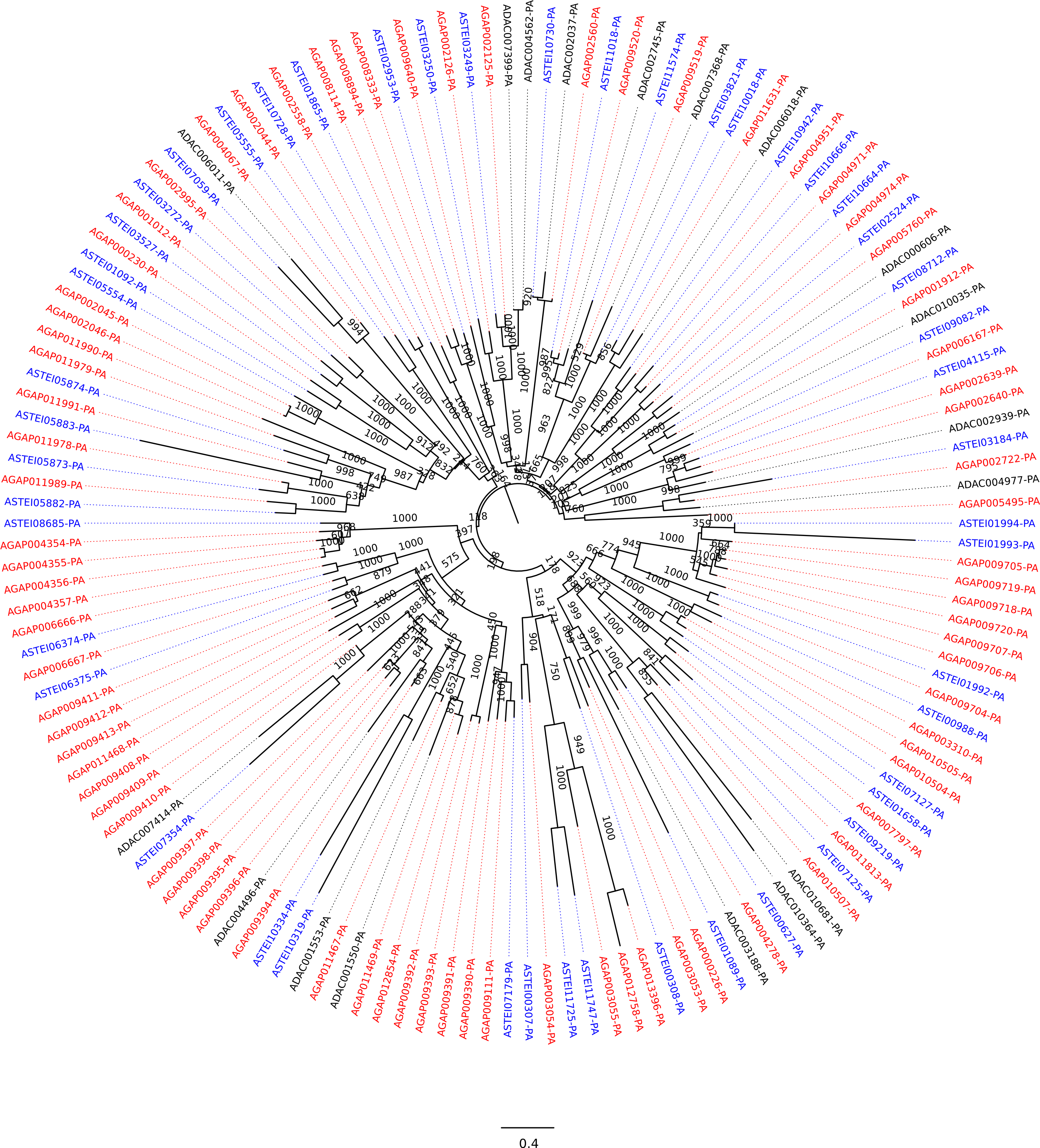


**C**
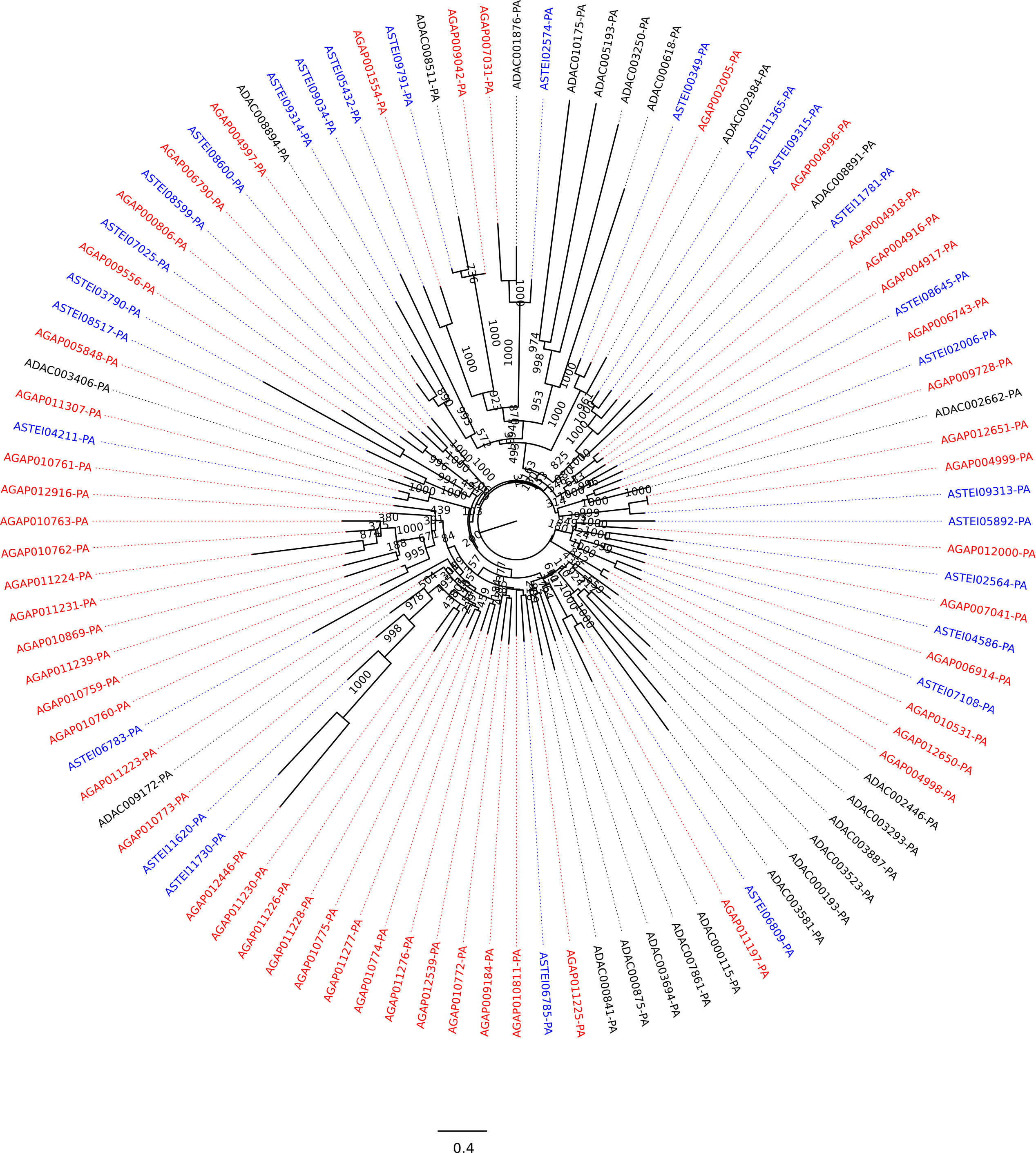


## Figure S4. Phylogenetic tree for *Anopheles* OBPs, OR and fibrinogen-related proteins.

Phylogenetic trees are constructed for genes families from *An. stephensi* (blue color), *An. gambiae* (red color), and *An. darlingi* (black color). For OBPs (A), strong one-to-one relationship was observed between *An. stephensi* and *An. gambiae*. For ORs (B) and fibrinogen-related proteins (C), there are more ‘expanded’ genes in *An. gambiae* than in *An. stephensi*.


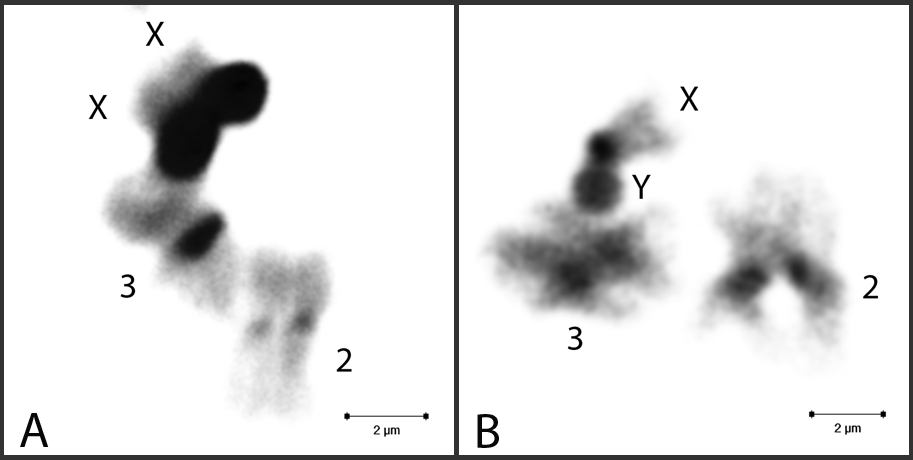


## Figure S5. Comparison of DAPI stained heterochromatin in mitotic chromosomes between *An. stephensi* and *An. gambiae*.

The *An. stephensi* chromosomes (A) exhibit much more heterochromatin than the chromosomes of *An. gambiae* (B). This difference is particularly evident in X chromosome where *An. stephensi* has substantially larger heterochromatin as compared with *An. gambiae*. The original color images were converted into grayscale and inverted for improved visibility of heterochromatin.


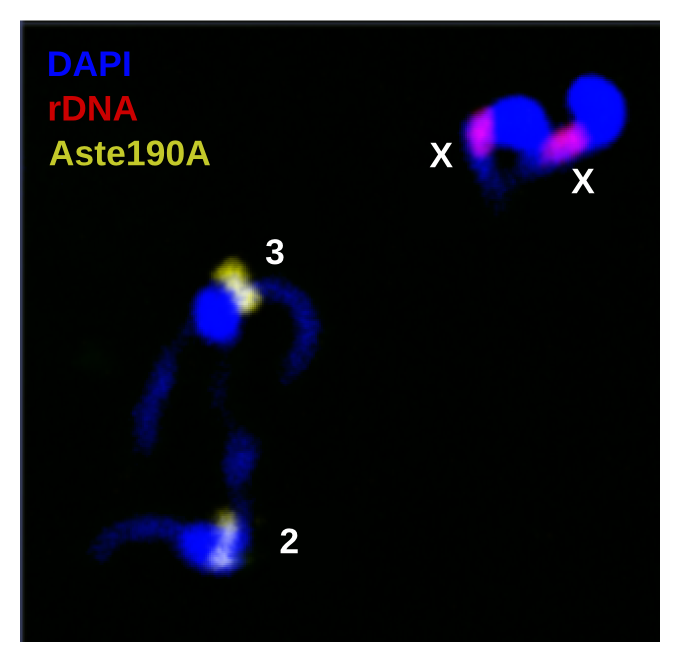


## Figure S6. FISH with Aste190A, rDNA, and DAPI on mitotic chromosomes.

The pattern of hybridization for satellite DNA Aste190A on mitotic sex chromosomes of *An. stephensi*. Aste190A hybridizes to centromere in autosomes while ribosomal DNA locus maps next to the heterochromatin band in sex chromosomes only.


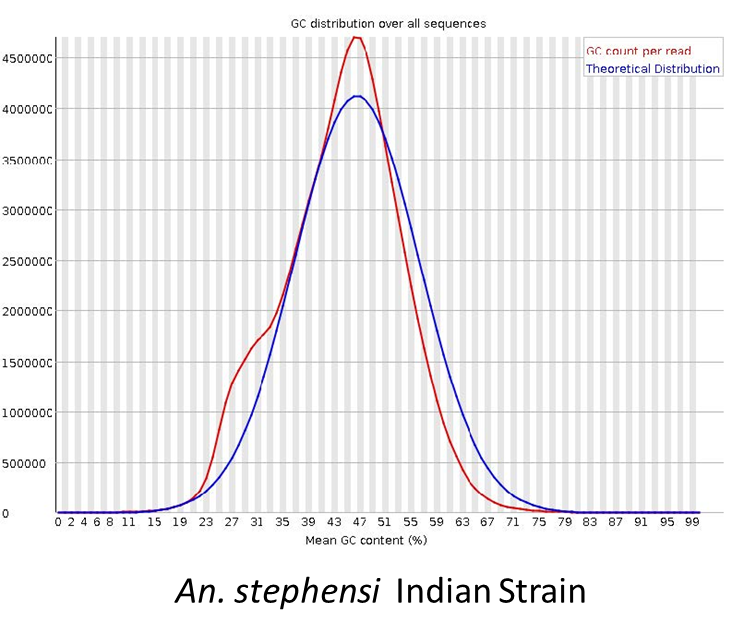


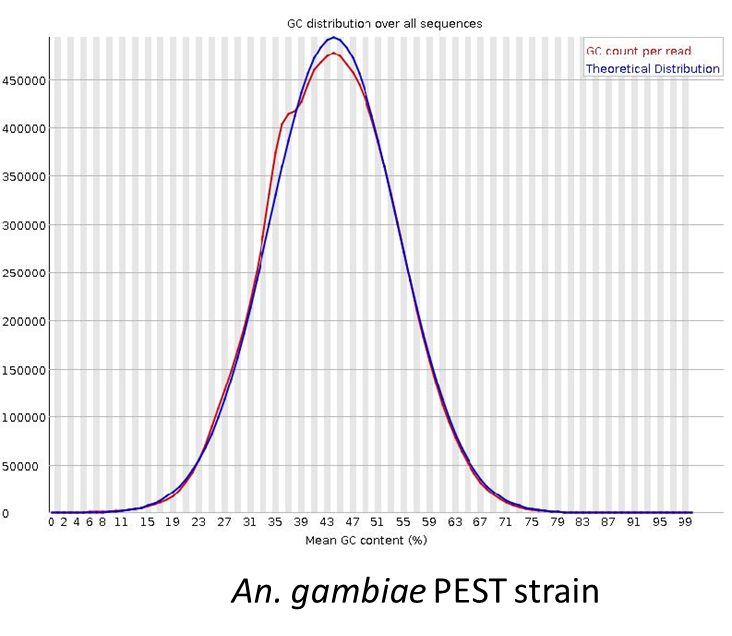


## Figure S7. The GC content in raw HiSeq reads of *An. stephensi* (left) and *An. gambiae* (right).

Red line represents GC content per sequence and blue line represents theoretical distribution. The results are obtained with the FastQC program (http://www.bioinformatics.babraham.ac.uk/projects/fastqc/). The majority of the reads from the ‘peak’ at the 26.7% mean GC content in *An. stephensi* corresponds to the Aste72A satellite DNA. The Y-axis shows the number of reads.

## Table S1. Data used for assembly.

| **Technology** | **Insert size** | **Reads (n)** | **Median length** | **Coverage**^a^ |
| --- | --- | --- | --- | --- |
| 454 | 3 kb | 1,632,796 | 341 | 2.2x |
| 454 | 8 kb | 2,503,762 | 339 | 3.4x |
| 454 | 20 kb | 2,211,050 | 194 | 1.7x |
| 454 | Shotgun | 8,143,060 | 395 | 12.1x |
| Illumina | 200 bp | 200,912,996 | 101 | 86.4x |
| PacBio^b^ | Shotgun | 753,589 | 1,295 | 5.2x |
| BAC-ends | 120 kb | 7,263 | 923 | 0.03x |
| ^a^Coverage based on estimated genome size of 235 Mbp.  ^b^PacBio was error corrected with the 454 reads. | | | | |

## Table S2. *An. stephensi* density/100 kb.

| **Chromosome arm** | **Genes** | **S/MARs** | **Microsatellites** | **Minisatellites** | **Satellites** |
| --- | --- | --- | --- | --- | --- |
| X | 5.62 | 2.37 | 7.88 | 7.9 | 0.13 |
| 2R | 6.38 | 1.59 | 0.77 | 0.94 | 0.06 |
| 3L | 6.83 | 2.25 | 0.88 | 0.92 | 0.06 |
| 3R | 5.63 | 2.39 | 0.76 | 0.76 | 0.1 |
| 2L | 6.1 | 2.76 | 0.75 | 0.96 | 0.06 |

## Table S3. *An. gambiae* density/100 kb.

| **Chromosome arm** | **Genes** | **S/MARs** | **Microsatellites** | **Minisatellites** | **Satellites** |
| --- | --- | --- | --- | --- | --- |
| X | 5.545 | 2.48 | 18.895 | 20.805 | 0.965 |
| 2R | 6.791 | 2.35 | 7.35 | 9.33 | 0.36 |
| 2L | 6.943 | 3.44 | 7.22 | 10.73 | 0.33 |
| 3R | 5.563 | 4.01 | 6.172 | 10.4 | 0.237 |
| 3L | 5.483 | 4.34 | 5.645 | 10.895 | 0.324 |

## Table S4. Synteny blocks and inversions between *An. stephensi* and *An. gambiae.*

| **Chromosome arm** | **Synteny blocks (n)** | **Inversions GRIMM (n)** |
| --- | --- | --- |
| X | 66 | 47 |
| 2R | 104 | 42 |
| 3L | 64 | 27 |
| 3R | 104 | 51 |
| 2L | 42 | 17 |

## Table S5. Inversion breaks/Mb between *An. stephensi* and *An. gambiae.*

| **Chromosome arm** | **Number of breaks (GRIMM)** | **Number of breaks (common polymorphic inversions)** |
| --- | --- | --- |
| X | 6.43 | 0.00 |
| 2R | 2.13 | 0.36 |
| 3L | 2.41 | 0.40 |
| 3R | 2.70 | 0.11 |
| 2L | 1.52 | 0.10 |

## Table S6. The ratio of the X chromosome evolution rate to the autosomal rate of rearrangement between *An. stephensi* and *An. gambiae*.

| **Chromosome arm** | **Chromosome size (Mb)** | **Inversions/Mb** | **Breaks/Mb/MY (Divergence time 30.4 MY)** |
| --- | --- | --- | --- |
| X | 14.619 | 3.22 | 0.106 |
| 2R | 39.497 | 1.06 | 0.035 |
| 3L | 22.409 | 1.20 | 0.040 |
| 3R | 37.708 | 1.35 | 0.044 |
| 2L | 22.342 | 0.76 | 0.025 |
| Total genome | 136.57 | 1.52 | 0.050 |

## Table S7. The ratio of the X chromosome evolution rate to the total rate of rearrangement in *Anopheles* and *Drosophila*.

| **Species pairs** | **All arms, breaks/MB/MY** | **X chromosome, breaks/MB/MY** | **X/All arms breaks** |
| --- | --- | --- | --- |
| *An. gambiae-An. stephensi* | 0.050 | 0.106 | 2.116 |
| *D. melanogaster-D. erecta* | 0.013 | 0.011 | 0.846 |
| *D. melanogaster-D. yakuba* | 0.020 | 0.022 | 1.100 |
| *D. melanogaster-D. ananassae* | 0.088 | 0.114 | 1.295 |
| *D. melanogaster-D. pseudoobscura* | 0.112 | 0.178 | 1.589 |
| *D. melanogaster-D. willistoni* | 0.171 | 0.220 | 1.287 |
| *D. melanogaster-D. virilis* | 0.138 | 0.155 | 1.123 |
| *D. melanogaster-D. mojavensis* | 0.137 | 0.149 | 1.088 |
| *D. melanogaster-D. grimshawi* | 0.159 | 0.199 | 1.252 |

## Table S8. Correlation of *An. stephensi* genes and rearrangements*.*

| ***An. stephensi*** | **Correlation w/ genes** |
| --- | --- |
| All arms w/GRIMM | -0.545 |
| Autosomes w/GRIMM | -0.131 |
| Common polymorphic inversions GRIMM (Autosomes) | 0.869 |

## Table S9. Correlation of *An. gambiae* genes and rearrangements*.*

| ***An. gambiae*** | **Correlation w/ Genes** |
| --- | --- |
| All arms w/GRIMM | -0.563 |
| Autosomes w/GRIMM | 0.144 |
| Common polymorphic inversions GRIMM (Autosomes) | 0.991 |

## Table S10. Correlation of *An. stephensi* S/MARs and rearrangements*.*

| ***An. stephensi*** | **Correlation w/ S/MARs** |
| --- | --- |
| All arms w/GRIMM | 0.057 |
| Autosomes w/GRIMM | -0.307 |
| Common polymorphic inversions | -0.716 |

## Table S11. Correlation of *An. gambiae* S/MARs and rearrangements*.*

| ***An. gambiae*** | **Correlation w/ S/MARs** |
| --- | --- |
| All Arms w/GRIMM | -0.449 |
| Autosomes w/GRIMM | -0.188 |
| Common polymorphic inversions | -0.799 |

## Table S12. Correlation of *An. stephensi* short tandem repeats and rearrangements*.*

| ***An. stephensi*** | **Microsatellites** | **Minisatellites** | **Satellites** |
| --- | --- | --- | --- |
| All arms w/GRIMM | 0.976 | 0.970 | 0.901 |
| Autosomes w/GRIMM | 0.353 | -0.797 | 0.679 |
| Common polymorphic inversions GRIMM (Autosomes) | 0.753 | 0.388 | -0.536 |

## Table S13. Correlation of *An. gambiae* short tandem repeats and rearrangements*.*

| ***An. gambiae*** | **Microsatellites** | **Minisatellites** | **Satellites** |
| --- | --- | --- | --- |
| All arms w/GRIMM | 0.976 | 0.938 | 0.938 |
| Autosomes w/GRIMM | 0.371 | -0.219 | -0.460 |
| Polymorphic inversions GRIMM (Autosomes) | 0.958 | -0.250 | 0.740 |

## Table S14. SNPs chromosomal distribution tables.

|  | **SNP counts** | **Total bp mapped to chromosomes** | **Frequency (counts/kbp)** |
| --- | --- | --- | --- |
| 2L | 51,425 | 22,341,925 | 2.3 |
| 2R | 108,010 | 39,497,041 | 2.7 |
| 3L | 56,277 | 22,408,440 | 2.5 |
| 3R | 95,596 | 37,780,138 | 2.5 |
| X | 8,443 | 14,903,228 | 0.57 |

## Table S15. tRNA tables.

| **Type** | **Number** |
| --- | --- |
| tRNAs Decoding Standard 20 AA | 337 |
| Selenocysteine tRNAs (TCA) | 1 |
| Possible suppressor tRNAs (CTA,TTA) | 0 |
| tRNAs with undetermined  or unknown isotypes | 6 |
| Predicted pseudogenes | 89 |
| Total tRNAs | 433 |

# References

1. Akbari OS, Antoshechkin I, Amrhein H, Williams B, Diloreto R, Sandler J, Hay BA: **The developmental transcriptome of the mosquito Aedes aegypti, an invasive species and major arbovirus vector.** *G3 (Bethesda)* 2013, **3:**1493–1509.

2. Marinotti O, Cerqueira GC, de Almeida LG, Ferro MI, Loreto EL, Zaha A, Teixeira SM, Wespiser AR, Almeida ESA, Schlindwein AD, Pacheco AC, Silva AL, Graveley BR, Walenz BP, Lima Bde A, Ribeiro CA, Nunes-Silva CG, de Carvalho CR, Soares CM, de Menezes CB, Matiolli C, Caffrey D, Araujo DA, de Oliveira DM, Golenbock D, Grisard EC, Fantinatti-Garboggini F, de Carvalho FM, Barcellos FG, Prosdocimi F, *et al*: **The genome of Anopheles darlingi, the main neotropical malaria vector.** *Nucleic Acids Res* 2013, **41:**7387–7400.

3. Zdobnov EM, von Mering C, Letunic I, Torrents D, Suyama M, Copley RR, Christophides GK, Thomasova D, Holt RA, Subramanian GM, Mueller HM, Dimopoulos G, Law JH, Wells MA, Birney E, Charlab R, Halpern AL, Kokoza E, Kraft CL, Lai Z, Lewis S, Louis C, Barillas-Mury C, Nusskern D, Rubin GM, Salzberg SL, Sutton GG, Topalis P, Wides R, Wincker P, *et al*: **Comparative genome and proteome analysis of Anopheles gambiae and Drosophila melanogaster.** *Science* 2002, **298:**149–159.

4. Bhutkar A, Schaeffer SW, Russo SM, Xu M, Smith TF, Gelbart WM: **Chromosomal rearrangement inferred from comparisons of 12 Drosophila genomes.** *Genetics* 2008, **179:**1657–1680.

5. Ranz JM, Maurin D, Chan YS, Von Grotthuss M, Hillier LW, Roote J, Ashburner M, Bergman CM: **Principles of genome evolution in the Drosophila melanogaster species group.** *PLoS Biology* 2007, **5:**1366–1381.

6. Schaeffer SW, Bhutkar A, McAllister BF, Matsuda M, Matzkin LM, O'Grady PM, Rohde C, Valente VLS, Aguadé M, Anderson WW, Edwards K, Garcia AC, Goodman J, Hartigan J, Kataoka E, Lapoint RT, Lozovsky ER, Machado CA, Noor MA, Papaceit M, Reed LK, Richards S, Rieger TT, Russo SM, Sato H, Segarra C, Smith DR, Smith TF, Strelets V, Tobari YN, *et al*: **Polytene chromosomal maps of 11 Drosophila species: the order of genomic scaffolds inferred from genetic and physical maps.** *Genetics* 2008, **179:**1601–1655.

7. Sharakhov IV, Serazin AC, Grushko OG, Dana A, Lobo N, Hillenmeyer ME, Westerman R, Romero-Severson J, Costantini C, Sagnon N, Collins FH, Besansky NJ: **Inversions and gene order shuffling in Anopheles gambiae and A. funestus.** *Science* 2002, **298:**182–185.

8. von Grotthuss M, Ashburner M, Ranz JM: **Fragile regions and not functional constraints predominate in shaping gene organization in the genus Drosophila.** *Genome Res* 2010, **20:**1084–1096.

9. Xia A, Sharakhova MV, Leman SC, Tu Z, Bailey JA, Smith CD, Sharakhov IV: **Genome landscape and evolutionary plasticity of chromosomes in malaria mosquitoes.** *PLoS ONE* 2010, **5:**e10592.

10. Kamali M, Marek PE, Peery A, Antonio-Nkondjio C, Ndo C, Tu Z, Simard F, Sharakhov IV: **Multigene phylogenetics reveals temporal diversification of major African malaria vectors.** *PLoS One* 2014, **9:**e93580.

11. Xia A, Sharakhova MV, Leman SC, Tu Z, Bailey JA, Smith CD, Sharakhov IV: **Genome landscape and evolutionary plasticity of chromosomes in malaria mosquitoes.** *PLoS One* 2010, **5:**e10592.

12. Ranz JM, Maurin D, Chan YS, von Grotthuss M, Hillier LW, Roote J, Ashburner M, Bergman CM: **Principles of genome evolution in the Drosophila melanogaster species group.** *PLoS Biol* 2007, **5:**e152.

13. Mahmood F, Sakai RK: **Inversion polymorphisms in natural populations of Anopheles stephensi.** *Can J Genet Cytol* 1984, **26:**538–546.

14. Coluzzi M, Sabatini A, della Torre A, Di Deco MA, Petrarca V: **A polytene chromosome analysis of the Anopheles gambiae species complex.** *Science* 2002, **298:**1415–1418.

15. Lobo NF, Sangare DM, Regier AA, Reidenbach KR, Bretz DA, Sharakhova MV, Emrich SJ, Traore SF, Costantini C, Besansky NJ, Collins FH: **Breakpoint structure of the Anopheles gambiae 2Rb chromosomal inversion.** *Malar J* 2010, **9:**293.

16. Coulibaly MB, Lobo NF, Fitzpatrick MC, Kern M, Grushko O, Thaner DV, Traoré SF, Collins FH, Besansky NJ: **Segmental Duplication Implicated in the Genesis of Inversion 2 Rj of Anopheles gambiae.** *PLoS One* 2007, **2:**e849.

17. Kamali M, Xia A, Tu Z, Sharakhov IV: **A new chromosomal phylogeny supports the repeated origin of vectorial capacity in malaria mosquitoes of the Anopheles gambiae complex.** *PLoS Pathog* 2012, **8:**e1002960.

18. Calvete O, González J, Betrán E, Ruiz A: **Segmental duplication, microinversion, and gene loss associated with a complex inversion breakpoint region in Drosophila.** *Mol Biol Evol* 2012, **29:**1875–1889.

19. Carver TJ, Rutherford KM, Berriman M, Rajandream M-A, Barrell BG, Parkhill J: **ACT: the Artemis Comparison Tool.** *Bioinformatics* 2005, **21:**3422–3423.
